# RING-finger protein 166 plays a novel pro-apoptotic role in neurotoxin-induced neurodegeneration via ubiquitination of XIAP

**DOI:** 10.1038/s41419-020-03145-x

**Published:** 2020-10-31

**Authors:** Chang-Ki Oh, Young Ki Choi, Ih-Yeon Hwang, Yeon Uk Ko, In Kwon Chung, Nuri Yun, Young J. Oh

**Affiliations:** 1 Department of Systems Biology, Yonsei University College of Life Science and Biotechnology, Seoul, 120-749 Republic of Korea; 2 Department of Molecular Medicine and Neuroscience Translational Center, The Scripps Research Institute, La Jolla, CA 92037 USA

**Keywords:** Cell death in the nervous system, Parkinson's disease

## Abstract

The dopaminergic neurotoxin, 6-hydroxydopamine (6-OHDA), has been widely utilized to establish experimental models of Parkinson disease and to reveal the critical molecules and pathway underlying neuronal death. The profile of gene expression changes following 6-OHDA treatment of MN9D dopaminergic neuronal cells was investigated using a TwinChip Mouse-7.4K microarray. Functional clustering of altered sets of genes identified RING-finger protein 166 (RNF166). RNF166 is composed of an N-terminal RING domain and C-terminal ubiquitin interaction motif. RNF166 localized in the cytosol and nucleus. At the tissue level, RNF166 was widely expressed in the central nervous system and peripheral organs. In the cerebral cortex, its expression decreased over time. In certain conditions, overexpression of RNF166 accelerates the naturally occurring neuronal death and 6-OHDA–induced MN9D cell death as determined by TUNEL and annexin-V staining, and caspase activation. Consequently, 6-OHDA–induced apoptotic cell death was attenuated in RNF166-knockdown cells. In an attempt to elucidate the mechanism underlying this pro-apoptotic activity, binding protein profiles were assessed using the yeast two-hybrid system. Among several potential binding candidates, RNF166 was shown to interact with the cytoplasmic X-linked inhibitor of apoptosis (XIAP), inducing ubiquitin-dependent degradation of XIAP and eventually accelerating caspase activation following 6-OHDA treatment. RNF166’s interaction with and resulting inhibition of the XIAP anti-caspase activity was further enhanced by XIAP-associated factor-1 (XAF-1). Consequently, depletion of RNF166 suppressed 6-OHDA–induced caspase activation and apoptotic cell death, which was reversed by XIAP knockdown. In summary, our data suggest that RNF166, a novel E3 ligase, plays a pro-apoptotic role via caspase activation in neuronal cells.

## Introduction

Parkinson disease (PD) is the second most common chronic neurodegenerative disorder. The neurologic hallmarks of PD include progressive loss of dopaminergic neurons in the substantia nigra pars compecta^1,2^. Although the etiology of PD remains unclear, considerable evidence suggests that multiple factors lead to PD, including oxidative stress, mitochondrial dysfunction, proteasome dysfunction, and inflammation^3–5^. Oxidative stress leads to the accumulation of reactive oxygen species (ROS) due to an imbalance between ROS production and the antioxidant defense system^6^. The accumulation of ROS inflicts neuronal damage that results in activation of the cell death pathway^7,8^. PD-inducing neurotoxins such as 6-OHDA and 1-methyl-4-phenyl-1,2,3,6-tetrahydropyridine were used to establish pathophysiologic PD models^9^. The mechanism underlying 6-OHDA–induced degeneration of dopaminergic neurons was primarily ascribed to abnormal ROS generation. Indeed, our laboratory showed that 6-OHDA indices generation of ROS such as superoxide anion and hydrogen peroxide, which trigger apoptotic signaling in primary cultures of mesencephalic and cortical neurons, MN9D dopaminergic neuronal cells, and rat brain models of PD^10–13^.

Ubiquitination plays a crucial role in regulating vital processes such as the cell cycle, cell signaling, and cell survival/death^14,15^. Ubiquitination is a major pathway in the elimination of accumulated toxic proteins and targeting of damaged proteins for degradation^16^. Several neurodegenerative disorders appear to share a common pathology typified by accumulation of abnormal protein aggregates^17^. Ubiquitination is a highly ordered multistep enzymatic process mediated by ubiquitin activating enzyme (E1), ubiquitin-conjugating enzyme (E2), and ubiquitin ligase (E3). Polyubiquitin chains added at lysine (K) residues alter the target protein’s function. The K48-linked chain is the best-characterized form of ubiquitination and leads to modification of protein substrates for proteasome degradation^18^. Multi-ubiquitin chains at least four ubiquitin molecules long must be attached to a lysine residue on the condemned protein to be recognized by the proteasome. In contrast, K63-linked ubiquitination are not associated with proteasomal degradation of the substrate protein. Instead, it leads to changes in protein subcellular localization, endocytic trafficking, inflammation, translation, and DNA repair^19^. The E3 ligases recognize and select protein substrates for ubiquitination and thus impart specificity to the process^20,21^.

The largest group of E3 ubiquitin ligases is the RING family. Several RING-finger–containing E3 ligases are implicated in disease processes^21^. E3 ligases confer specificity to ubiquitination by recognizing target substrates and mediating the transfer of ubiquitin to the substrate^18^. These modifications exert diverse effects on substrates, ranging from proteasome-dependent proteolysis to modulation of protein function. For example, parkin possesses two RING-finger domains and an in-between-RING region domain that functions in a multiprotein ubiquitin ligase complex^22^. Many disease-causing mutations in parkin apparently lead to loss of function via decreased catalytic activity, aberrant ubiquitination, or impaired proteasomal degradation^23,24^. RING-finger protein 166 (RNF166) is a member of the RING-domain E3 ubiquitin ligase family. Although only reports have addressed its functional role to date, roles for RNF166 in RNA virus–induced innate immune responses and antibacterial autophagy were reported^25,26^. RNF166 positively regulates RNA virus–triggered interferon-β production by enhancing the ubiquitination of TRAF3/6^25^. RNF166 regulates xenophagy by interacting with the autophagy network and recruiting ubiquitin and the autophagy adaptors to invading bacteria^26^. However, no studies have elucidated the role of RNF166 in the pathophysiology of the central nervous system (CNS).

Here, we attempted to classify RING-domain genes identified via microarray analysis using inputs obtained from 6-OHDA-treated cells. We then attempted to identify a novel E3 ligase from the pool of identified candidates exhibiting altered expression. Further study focused on clarifying the potential role of RNF166 in naturally occurring and 6-OHDA-induced neuronal cell death. We demonstrate that RNF166 protein is widely distributed in the CNS from the embryonic to adult stages, and its levels decrease over time during development of the cerebral cortex. Independent biochemical and genetic approaches revealed that RNF166 exerts pro-apoptotic effects via ubiquitin-dependent degradation of XIAP and subsequent over-activation of caspase-dependent cell death processes following 6-OHDA treatment.

## Results

### Expression of RNF166 is altered during 6-OHDA-induced neuronal cell death

We previously conducted TwinChip-based microarray analyses using samples of 6-OHDA-treated MN9D cells^27^. At present, these microarray data were clustered using keywords included the RING domain (*q*-value ≤ 0.05) to identify novel factor(s) that play critical role(s) in regulating neuronal death via E3 ligase activity. A heatmap of 13 clustered genes was produced using TreeView software (version 1.60) (Supplementary Fig. 1A)^28^. The RNF166 gene was selected from the clustered genes because its expression was augmented considerably during 6-OHDA–induced neurodegeneration. To validate the microarray data, RNF166 mRNA levels were examined by quantitative polymerase chain reaction (qPCR) using RNA extracts isolated from MN9D cells treated with 100 µM 6-OHDA (Supplementary Fig. 1B). The expression pattern was similar to that of the microarray analysis. Searches of known databases indicated that RNF166 is composed of an N-terminal C3HC4-type RING domain, zinc finger C2H2 domain, and a C-terminal ubiquitin interaction motif (UIM) (Supplementary Fig. 2A). Moreover, the sequence homology was conserved across different species (Supplementary Fig. 2B).

### Tissue and cellular distribution of RNF166

**Fig. 1:**
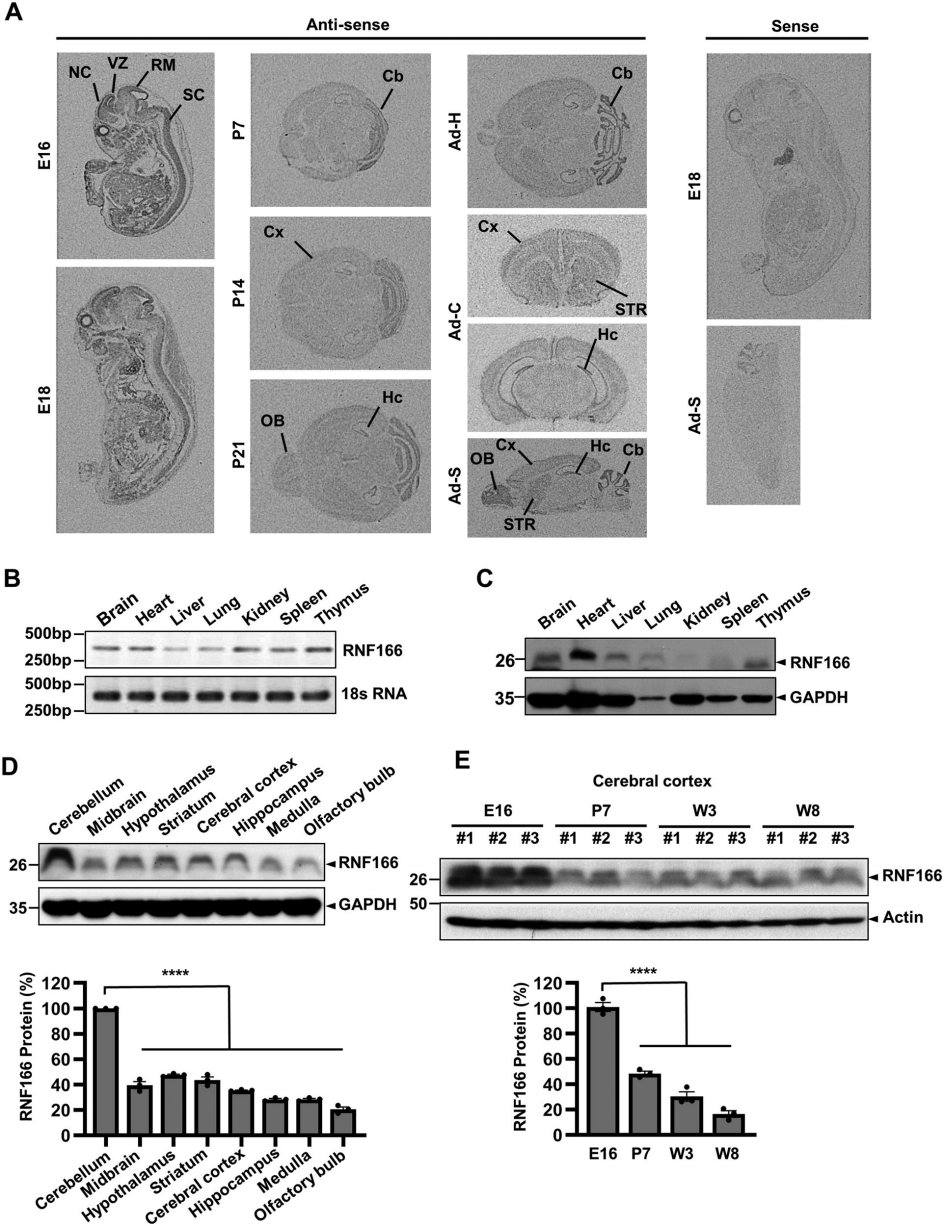
Temporal and spatial expression pattern of RNF166 mRNA and protein in various tissues. **a** In situ hybridization assay was performed with [^35^S]-labeled RNA probes encoding antisense sequences of mouse RNF166 encompassing 1~500 bp. Various planes of section were examined to better visualize the expression pattern of RNF166 mRNA at the indicated stages of development: sagittal sections of the whole body at E16 and E18; horizontal sections of the mouse brain at postnatal day 7 (P7), P14, and P21; horizontal (H), coronal (C), and sagittal (S) sections of 2-month-old adult brain. For negative controls, [^35^S]-labeled RNA probes encoding sense sequences of RNF166 were hybridized with midsagittal sections of E18 embryos and adult mouse brain. The following abbreviations are used from left to right of the antisense panel: NC neopallial cortex, VZ ventricular zone, RM roof of midbrain, SC spinal cord, Cb cerebellum, Cx cerebral cortex, OB olfactory bulb, Hc hippocampus, STR striatum. Photomicrographs represent data acquired from RNA probe #1. Virtually identical patterns were obtained using RNA probes #2 and #3. **b** Total RNA prepared from various tissues of adult mice were subjected to RT-PCR. As an internal control, universal 18S primers were included in the same reaction mixtures. **c** Tissue lysates were prepared from the various body parts of adult mice and subjected to immunoblotting using anti-RNF166 antibody. Anti–glyceraldehyde-3-phosphate dehydrogenase (GAPDH) was used as a loading control. **d** Tissue lysates obtained from various brain parts of adult rats were subjected to immunoblotting using anti-RNF166 antibody. Densitometric values of RNF166 normalized to GAPDH are expressed as a percentage of that in the cerebellum (value = 100%). Bars represent the mean + SEM of three independent experiments. ****p < 0.0001. **e** Temporal expression levels of RNF166 determined using tissue lysates obtained from rat cerebral cortex at E16, P7, postnatal week 3 (W3), and W8. Densitometric values of RNF166 signals were normalized against actin and are expressed as percentage relative to that in E16 cortices (value = 100%). Bars represent the mean + SEM from three independent experiments. ****p < 0.0001.

The expression pattern of RNF166 mRNA in mouse embryo and adult brain was evaluated by in situ hybridization on embryonic days 16 and 18, postnatal days 7, 14, and 21, and adult stages. During the embryonic stages, RNF166 mRNA was highly expressed in the neopallial cortex, midbrain roof, ventricular zone, and spinal cord (Fig. 1a). At the postnatal to adult stages, higher RNF166 mRNA expression was observed in the olfactory bulb, cerebral cortex, cerebellum, striatum, and hippocampus. RNF166 mRNA expression levels in the whole body were assessed by revers transcription (RT)-PCR using total RNA extracts prepared from the mouse tissues. RNF166 mRNA was present in both neural and non-neural tissues, including the heart, liver, lung, kidney, spleen, and thymus (Fig. 1b). Consistent with the widespread expression of RNF166 mRNA, immunoblot analyses revealed RNF166 protein expression in various organs (Fig. 1c) and brain regions (Fig. 1d). The cerebellum exhibited the highest expression. The expression levels of RNF166 during development and aging was evaluated by monitoring the temporal expression of RNF166 in the cerebral cortex. Immunoblotting indicated higher expression in the embryonic age (E16), which significantly decreased from the postnatal to adult stages (Fig. 1e). The cellular localization of endogenous and green fluorescent protein (GFP)-tagged RNF166 was determined in MN9D cells. Although it was not understood reason behind the somewhat different localization pattern of C-terminal and N-terminal empty GFP vector, fluorescence images indicated both N- and C-terminal GFP-tagged RNF166 in the cytoplasm and nucleus, regardless of fixation/permeabilization reagents used (Supplementary Fig. 3A). A similar distribution pattern was observed in MN9D cells transfected with RNF166-V5 or Flag-RNF166 (Supplementary Fig. 3B). Consistent with these data, immunoblotting using equal amounts of protein from each fraction revealed endogenous RNF166 in both the cytosolic and nuclear fractions (Supplementary Fig. 3C). We did not further attempt to directly compare the relative expression levels of RNF166 present in both the cytosol and the nucleus.

### Pro-apoptotic role of RNF166 in two independent models of naturally occurring neuronal death

**Fig. 2:**
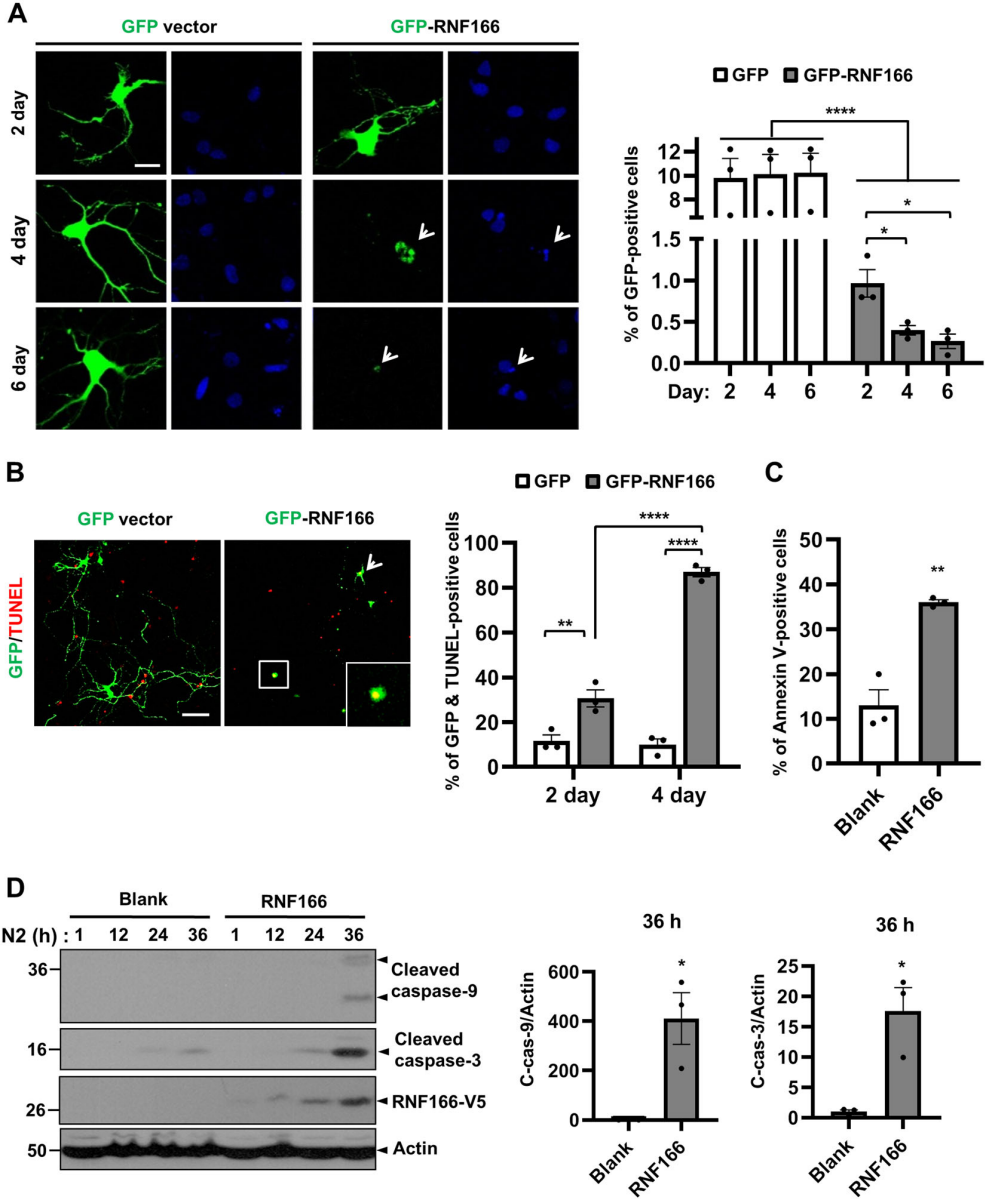
Pro-apoptotic function of RNF166 in naturally occurring neuronal death. **a**–**b** Primary hippocampal neurons at DIV 1 were transiently transfected with GFP- or GFP-tagged RNF166 using Lipofectamine 2000 and further cultivated for the indicated time periods. **a** At the indicated time periods, cells were counterstained with 1 mg/mL Hoechst 33258 and then examined under confocal microscopy. Representative fluorescence images are shown. Arrowheads indicate typical dying cells with no apparent GFP signal or fragmented GFP staining. Scale bar, 50 µm. GFP-positive cells were counted by flow cytometry analysis. Values are expressed as the number of GFP-positive cells at the indicated time period after transfection. Each value represents the mean + SEM of at least three separate experiments. ****p < 0.0001, *p < 0.05 (RNF166; 2 day vs. 4 day), and *p < 0.05 (RNF166; 2 days vs. 6 days). **b** At 4 days post-transfection, cells were processed for TUNEL staining and examined under confocal microscopy. Arrowheads indicate typical GFP- and TUNEL-positive cells. Inset shows an enlarged view of the cell boxed in white in the merged image. Scale bar, 100 µm. In each of 40 randomly selected microscopic areas, the number of TUNEL-positive cells among GFP-positive cells was determined. Each value represents the mean + SEM of at least three separate experiments. ****p < 0.0001, **p < 0.01, *p < 0.05. **c** MN9D cells transfected with GFP-vector encoding/not encoding mouse the RNF166 cDNA sequence were cultivated in serum-free basal N2 medium. At 36 h, MN9D cells were stained with annexin V for 30 min at room temperature. Subsequently, stained cells were subjected to flow cytometry analysis. Data represent the mean + SEM of at least three separate experiments. **p < 0.01, *p < 0.05. **d** At the indicated time periods, cell lysates were subjected to immunoblotting using anti-cleaved caspase-3 and -9 antibodies. Anti-actin antibody was used as a loading control. After normalization against actin, densitometric values of cleaved forms of caspase-9 (C-cas-9) and capsase-3 (C-cas-3) were expressed as fold-change relative to that of the untreated control (value = 1). Bars represent the mean + SEM from three independent experiments. *p < 0.05.

The temporal expression pattern of RNF166 in the cerebral cortex led us to examine the functional role of RNF166 in naturally occurring cell death. Primary hippocampal neurons were transiently transfected with GFP-tagged vector with or without mouse RNF166 cDNA sequences. GFP-positive neurons extended neurites in control mock-transfected cells, whereas fragmentation of both soma and neurites and disappearance of GFP-positivity were observed in GFP-RNF166-expressing cells (Fig. 2a). Flow cytometry indicated significant loss of GFP-positivity starting 2 days post-transfection and continuing to 6 days post-transfection. We then investigated whether the loss of GFP signals in GFP-RNF166–transfected cells was associated with apoptosis. Hippocampal neurons were transiently transfected with GFP alone or GFP-RNF166 and subjected to terminal deoxynucleotidyl transferase dUTP nick-end labeling (TUNEL). The number of TUNEL-positive cells among GFP-positive cells at 2 days was higher, and a greater number of apoptotic cells was observed at 4 days post-transfection (Fig. 2b). We determined that RNF166 exhibited a pro-apoptotic effect in NeuN-positive hippocampal neurons (Supplementary Fig. 4). In MN9D cells cultured in serum-free N2 medium for 36 h, the number of annexin V-positive cells increased in the GFP-RNF166–transfected group (Fig. 2c). Immunoblotting revealed prominent activation of caspases 3 and 9, in RNF166-expressing MN9D cells (Fig. 2d), indicating a pro-apoptotic role for RNF166 during serum-deprivation-induced neuronal death as well.

### RNF166 exhibits E3 ubiquitin ligase activity

**Fig. 3:**
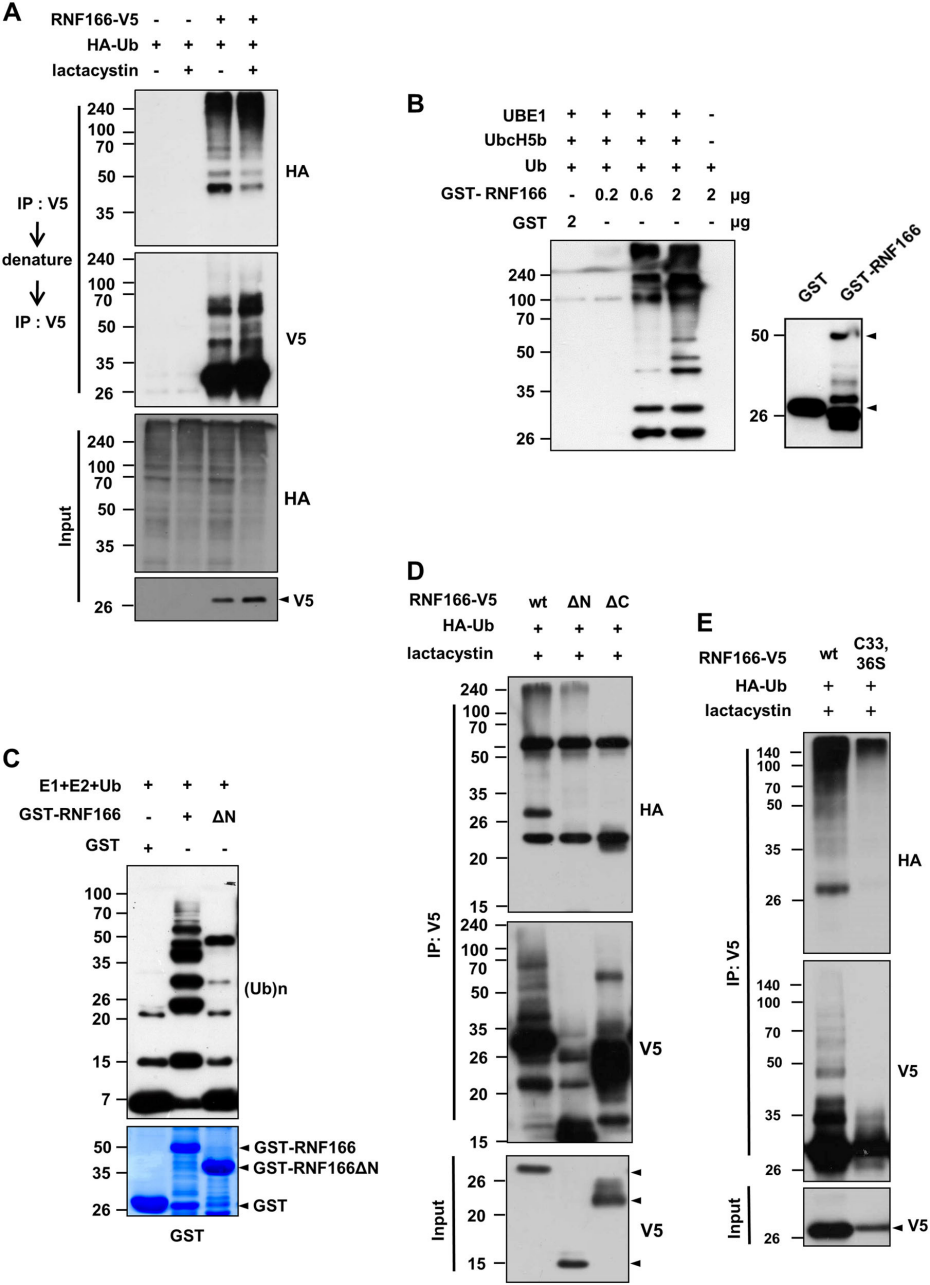
RING domain of RNF166 is important for its E3 ubiquitin ligase activity, as determined using cell-based and *in vitro* ubiquitination assays. **a** MN9D cells were transiently transfected with RNF166-V5 and Flag-ubiquitin (Ub). After transfection for 24 h, cells were cultured in the presence or absence of 2.5 mM lactacystin for an additional 24 h. Cell lysates were subjected to cell-based ubiquitination assay by immunoprecipitation with anti-V5 antibody under denature conditions followed by immunoblotting using anti-HA or anti-V5 antibody. Input was immunoprobed using anti-V5 or anti-HA antibody. **b**, **c** In vitro ubiquitination assay was conducted using purified GST or GST-RNF166. **b** Increasing doses of GST-RNF166 were incubated with E1 (UBE1), E2 (UbcH5b), and Ub along with ATP. Immunoblotting was performed using anti-Ub (left panel) or anti-GST antibody (right panel). **c** Purified GST-tagged RNF166 or its N-terminal deletion mutant (ΔN) were processed for in vitro ubiquitination assays. Immunoblots were probed with anti-Ub antibody. Bottom panel represents the Coomassie-stained gel. **d** MN9D cells were transiently transfected with the indicated combination of vectors. At 24 h post-transfection, cells were cultivated for an additional 24 h in the presence of 2.5 mM lactacystin. Cell lysates were subjected to a cell-based ubiquitination assay by immunoprecipitation with anti-V5 antibody under denaturing conditions followed by immunoblotting using anti-HA and anti-V5 antibody. **e** RNF166-V5– and RNF166C33,36S-V5–transfected MN9D cells were subjected to a cell-based ubiquitination assay as described in **d**.

Many RING-domain-containing proteins are ubiquitinated via an auto-catalytic process indicative of E3 ligase activity^18^. To determine whether RNF166 exerts E3 ligase activity, an auto-ubiquitination assay was performed using MN9D cells transiently transfected with hemagglutinin (HA)-ubiquitin and RNF166-V5. A cell-based auto-ubiquitination assay was performed by immunoprecipitation in conjunction with a denaturation step to eliminate signals from contaminant polyubiquitinated proteins other than RNF166^29,30^. Polyubiquitin smears appeared in samples of cells transfected with RNF166 (Fig. 3a). The extent of polyubiquitination of RNF166 increased in the presence of lactacystin, a proteasome inhibitor. To confirm the E3 ligase activity of RNF166, an in vitro ubiquitination assay was performed using purified GST and GST-RNF166. Consistent with cell-based auto-ubiquitination assay results, GST-RNF166 exhibited auto-ubiquitination in reaction mixtures containing ubiquitin, E1, and E2 (Fig. 3b). This reaction was dose dependent, as increasing GST-RNF166 concentrations generated more-prominent smearing of polyubiquitinated proteins. An RNF166 N-terminal RING-domain deletion mutant exhibited reduced polyubiquitination (Fig. 3c). To further confirm the critical RNF166 domain required for E3 ligase activity, MN9D cells were transfected with various combinations of vectors (Supplementary Fig. 2A). The extent of polyubiquitination decreased in cells transfected with the N-terminal RING-domain deletion vector and was more dramatically down-regulated in cells transfected with the C-terminal UIM deletion vector (Fig. 3d). In a double-point mutant (C33, 36S) of the RING domain, RNF166-mediated generation of polyubiquitination bands in MN9D cells was depleted (Fig. 3e), indicating that the N-terminal RING domain of RNF166 is important for E3 ligase activity–dependent ubiquitination.

### RNF166-XIAP physical interaction is enhanced by XAF-1

**Fig. 4:**
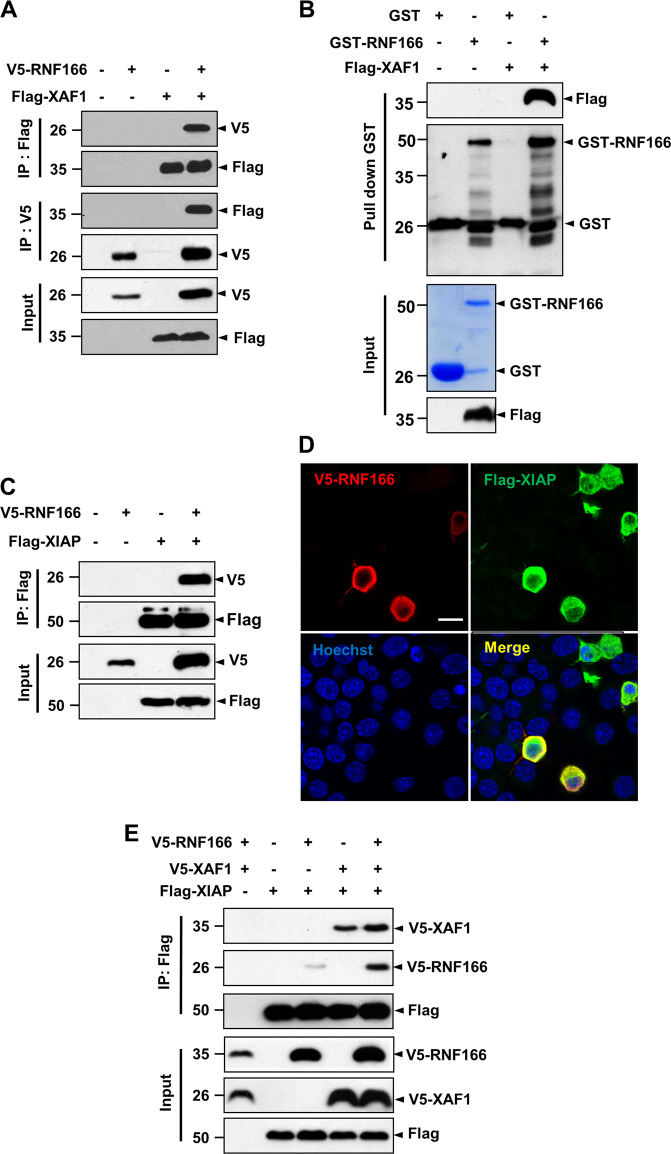
RNF166 physically interacts with XIAP, and this interaction is enhanced by XAF-1. **a** Co-immunoprecipitation assay was performed using HEK293 cells transfected with vector containing V5-RNF166 or Flag-XAF-1 or in combination. Cell lysates were subjected to immunoprecipitation with anti-Flag M2 affinity gel or anti-V5 antibody followed by immunoblotting using the indicated antibody. **b** GST pull-down assay was performed using purified GST- or GST-RNF166. As a source of XAF-1, cell lysates obtained from HEK293 cells transfected with Flag-XAF-1 were added to the reaction mixtures. After GST pull-down, immunoblotting was carried out using anti-Flag (upper panel) or anti-GST antibody (2^nd^ panel). Coomassie Blue-stained gels and anti-Flag–immunoprobed blots are shown (bottom two panels, respectively). **c** For co-immunoprecipitation assays, HEK293 cells were transfected with a eukaryotic expression vector harboring V5-RNF166 and/or Flag-XIAP. Cell lysates were immunoprecipitated with anti-Flag M2 affinity gel followed by immunoblotting using anti-V5 or anti-Flag antibody. **d** MN9D cells transiently expressing V5-RNF166 and Flag-XIAP were fixed with 4% paraformaldehyde and incubated with anti-mouse V5 and anti-rabbit Flag antibody followed by incubation with Alexa 568-conjugated goat anti-mouse IgG or Alexa 488-conjugated goat anti-rabbit IgG. Nuclei were counterstained with Hoechst 33258. Cells were then examined under a confocal microscope. Scale bar, 20 µm. **e** HEK293 cells were transfected for 48 h with the indicated combinations of eukaryotic expression vectors encoding Flag-XIAP, V5-RNF166, or V5-XAF-1. Coimmunoprecipitations were performed using anti-Flag M2 affinity gel, and immune complexes were subjected to immunoblotting using the indicated antibodies.

To elucidate the mechanism underlying the pro-apoptotic function of RNF166 in conjunction with its E3 ligase activity, we identified RNF166 interaction partners using yeast two-hybrid screens with full-length mouse RNF166 as bait. A clone (Supplementary Table 1) encoding XAF-1 was noteworthy, as XAF-1 is known to interact with XIAP^31^. To confirm the interaction between RNF166 and XAF-1, reciprocal co-immunoprecipitation was performed using human embryonic kidney 293 (HEK293) cells transfected with V5-RNF166 and Flag-XAF-1 alone or in combination. This assay confirmed that RNF166 interacts with XAF-1 (Fig. 4a). Consistent with these results, a GST pull-down assay indicated that RNF166 directly interacts with XAF-1 (Fig. 4b). We then examined whether RNF166 regulates XAF-1 stability via ubiquitination. In a cell-based ubiquitination assay, RNF166 did not clearly ubiquitinate XAF-1 (Supplementary Fig. 5A). Consistent with a previous report^32^, RNF166 upregulated XAF-1 expression in both the control and 6-OHDA-treated group (Supplementary Fig. 5B). As XAF-1 suppresses the anti-caspase activity of XIAP via binding^33^, we examined whether RNF166 associates with XIAP and subsequently ubiquitinates XIAP. In co-immunoprecipitation and immunofluorescence analyses, RNF166 interacted and co-localized with XIAP (Fig. 4c, d, respectively). The RNF166 domain necessary for interaction with XIAP was mapped using co-immunoprecipitation. XIAP interacted with RNF166 regardless of deletion of the RING or UIM domains (Supplementary Fig. 6A). However, only wild-type XIAP effectively bound to RNF166 (Supplementary Fig. 6B), suggesting that RNF166 interacts with the RING domain of XIAP. Interestingly, the interaction between RNF166 and XIAP increased in cells co-expressing XAF-1 (Fig. 4e), suggesting that the interaction between RNF166 and XIAP is enhanced by XAF-1.

### RNF166 is a novel ubiquitin E3 ligase for XIAP

**Fig. 5:**
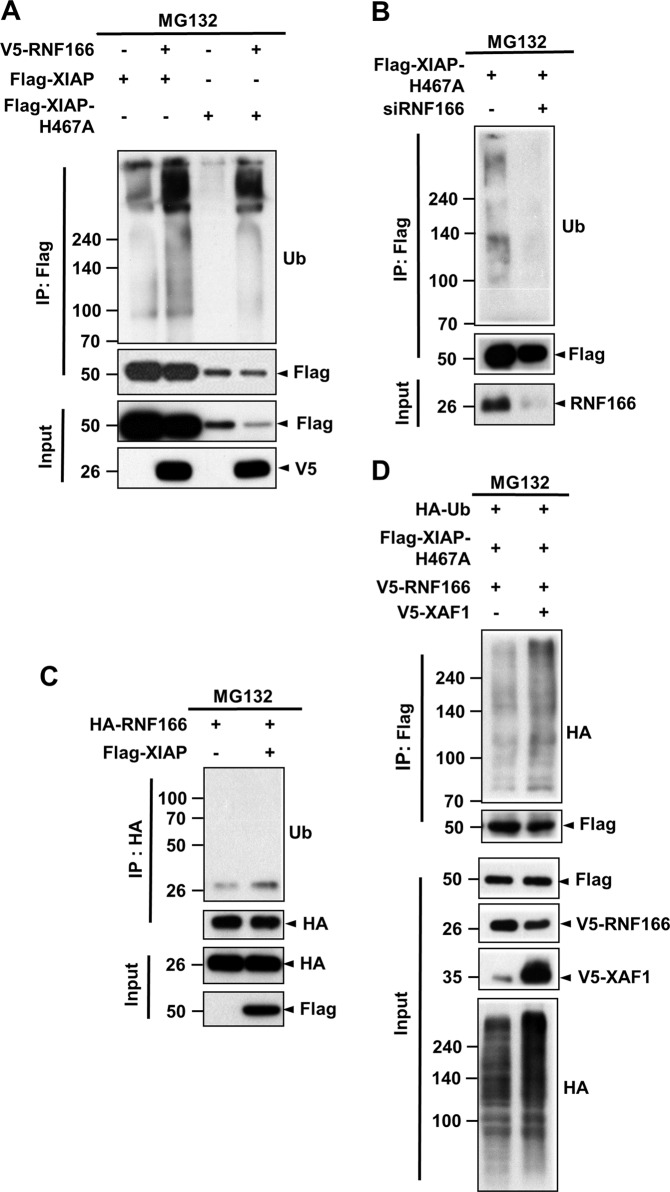
RNF166 is a novel E3 ligase for polyubiquitination of XIAP. **a**–**d** HEK293 cells were transiently transfected with the indicated combination of vectors encoding V5-RNF166, Flag-XIAP, or Flag-XIAP H467A. Cells were treated with 10 µM MG132 for 4 h prior to preparing cell lysates. **a** At 48 h after transfection, cell lysates were prepared and subjected to denaturing immunoprecipitation using anti-Flag M2 affinity gel. Polyubiquitination bands of XIAP were detected by immunoblotting using an anti-Ub antibody. **b** For knockdown experiments, cells were transfected with Flag-XIAP H467A plus scrambled control (−) or RNF166 siRNA. To measure the extent of polyubiquitination of XIAP in RNF166-depeleted cells, cell lysates were processed as described in **a**. **c** To determine whether XIAP ubiquitinates RNF166, cells were transiently transfected with HA-RNF166 alone or in combination with Flag-XIAP. Cell lysates were subjected to denaturing immunoprecipitation using anti-HA antibody followed by immunoblotting using anti-Ub antibody as described in **a**. **d** Lysates obtained from cells transiently transfected with the indicated combination of vectors were subjected to denaturing immunoprecipitation with anti-Flag M2 affinity gel. Polyubiquitination of XIAP was confirmed by immunoblot analysis using an anti-HA antibody.

A cell-based ubiquitination assay indicated that RNF166 overexpression led to increased polyubiquitinated XIAP bands (Fig. 5a; lane 1 vs. lane 2). Similarly, RNF166 overexpression also enhanced polyubiquitination of the XIAP H467A mutant, which exhibits loss of intrinsic E3 ligase activity and self-ubiquitination (Fig. 5a; lanes 3 and 4)^30^, indicating that the appearance of smeared XIAP bands resulted primarily from RNF166-mediated polyubiquitination of XIAP. Consistent with these data, transient knockdown using siRNF166 RNA decreased XIAP polyubiquitination (Fig. 5b). As expected, overexpression or knockdown of RNF166 resulted in decrease or increase of endogenous XIAP, respectively (Supplementary Fig. 7A, B). As XIAP is a RING-type E3 ligase, whether XIAP directly ubiquitinates RNF166 was also examined. However, XIAP did not polyubiquitinate RNF166 (Fig. 5c). In consistency with co-immunoprecipitation data demonstrating that XAF-1 enhances the RNF166/XIAP interaction (Fig. 4e), we demonstrated that XIAP polyubiquitination bands increased in cells co-expressing RNF166 and XAF-1 (Fig. 5d), indicating that XAF-1 enhances the RNF166/XIAP interaction and subsequent XIAP ubiquitination by RNF166.

### RNF166 upregulates apoptosis by downregulating XIAP in 6-OHDA–induced cell death

**Fig. 6:**
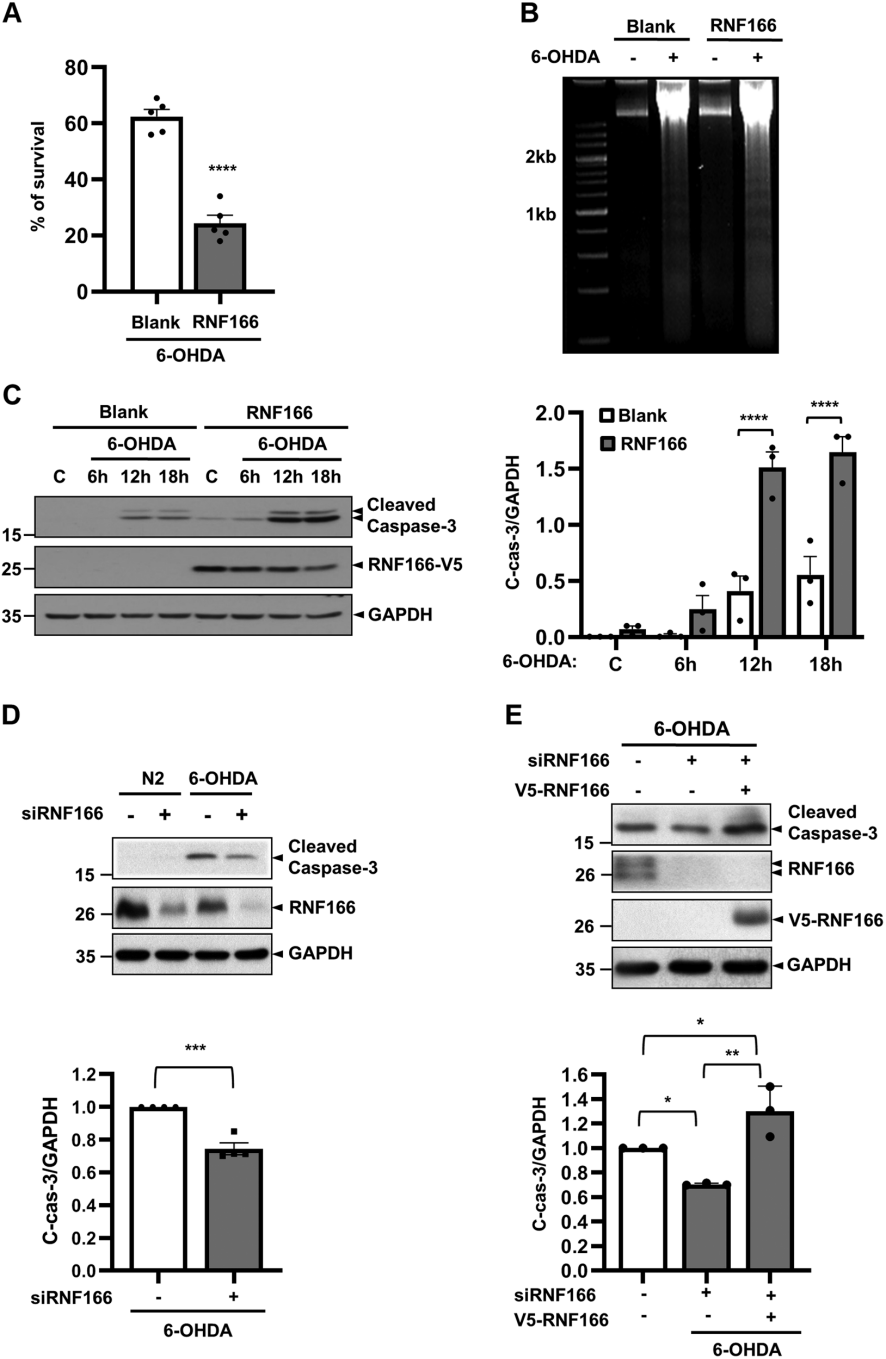
RNF166 accelerates apoptosis induced by 6-OHDA. **a**–**c** MN9D cells transfected with blank vector or vector encoding the mouse RNF166 cDNA sequence were treated with 100 µM 6-OHDA for the indicated time periods. **a** Following treatment with 6-OHDA for 18 h, the rate of cell survival was determined using an MTT reduction assay. Values are expressed as a percentage of untreated control cells (100%). Bars represent the mean + SEM of five independent experiments in triplicate. ****p < 0.0001, *p < 0.05. **b** Soluble DNA was extracted from mock-transfected or RNF166-transfected cells in lysis buffer containing proteinase K and subjected to electrophoresis on 1.5% agarose gel. Subsequently, gels were stained with ethidium bromide and then photographed on an UV transilluminator. **c** Cell lysates obtained from each group at the indicated time periods after 6-OHDA treatment were subjected to immunoblotting using the indicated antibodies, including anti-cleaved caspase-3 antibody. Densitometric values of C-cas-3 are expressed as a relative fold-change after normalization against GAPDH. Bars represent the mean + SEM from three independent experiments. ****p < 0.0001, *p < 0.05. **d** MN9D cells were transiently transfected with RNF166 siRNA and exposed to 100 µM 6-OHDA for 12 h. Cell lysates were subjected to immunoblotting using anti-cleaved caspase-3 and anti-RNF166 antibodies. Densitometric values of C-cas-3 are expressed as fold-change relative to control siRNA–transfected cells after normalization against GAPDH (value = 1). Bars represent the mean + SEM from three independent experiments. Quantitative analysis was performed as described above. ***p < 0.001, *p < 0.05. **e** For rescue experiments, MN9D cells transfected with RNF166 siRNA for 48 h were further transfected with or without vector encoding V5-RNF166 for an additional 24 h. Cells were then treated with 100 µM 6-OHDA for 12 h. Cell lysates were subjected to immunoblotting using anti-cleaved caspase-3 or anti-RNF166 antibody. Densitometric values of C-cas-3 are expressed as fold-change relative to the non-transfected control cells after normalization against GAPDH (value = 1). Bars represent the mean + SEM from the independent experiments. **p < 0.01; *p < 0.05.

We previously established that caspase-dependent cell death occurs in various neuronal cells exposed to 6-OHDA^11–13^. Therefore, we examined whether the pro-cell death function of RNF166 involves acceleration of caspase activation in a 6-OHDA-induced model. 3-(4,5-Dimethylthiazol-2-yl)-2,5-diphenyltetrazolium bromide (MTT) reduction assays demonstrated that approximately 62% of MN9D cells transfected with empty vector survived, whereas only 24% of RNF166-overexpressing MN9D cells survived following treatment with 6-OHDA for 18 h (Fig. 6a). The extent of DNA fragmentation and level of cleaved caspase-3 indicative of apoptosis were greater in RNF166-overexpressing cells than empty vector–transfected cells (Fig. 6b, c, respectively). To further confirm its pro-apoptotic role, endogenous RNF166 was depleted in MN9D cells using small hairpin RNA (shRNA)-mediated stable knockdown via lentiviral transduction (Supplementary Fig. 8A, B). MTT reduction assays demonstrated that RNF166 knockdown enhanced the viability of two stable cell lines following 6-OHDA treatment (Supplementary Fig. 8C). Immunoblotting indicated that 6-OHDA–induced generation of the cleaved caspase-3 decreased in RNF166-knockdown cells (Supplementary Fig. 8D). Similarly, caspase-3 activation declined significantly following 6-OHDA treatment in MN9D cells transiently depleted using siRNF166 RNA as compared to mock-transfected cells (Fig. 6d). Re-introduction of RNF166 restored cleaved caspase-3 to the peak level following 6-OHDA treatment (Fig. 6e).

**Fig. 7:**
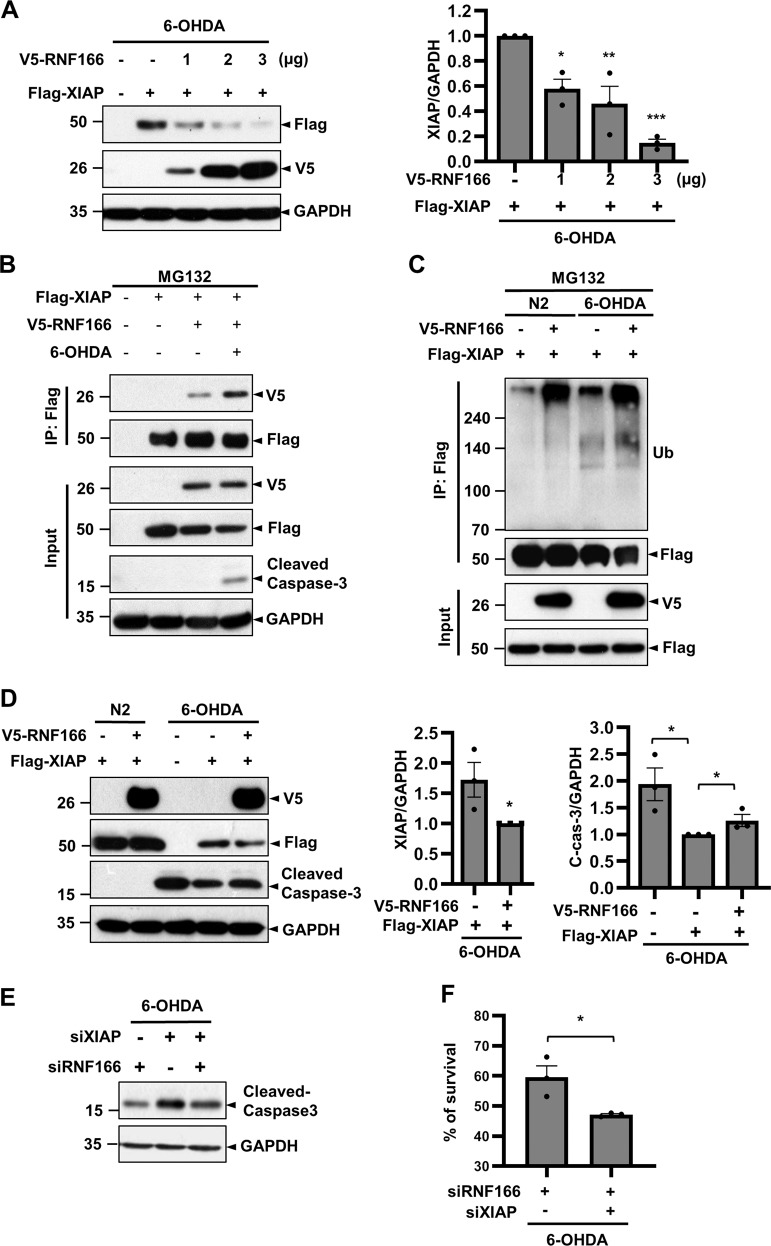
RNF166 enhances apoptosis by downregulation of XIAP expression and concomitant activation of caspase-3 following 6-OHDA treatment. **a** MN9D cells were transiently transfected with vector encoding Flag-XIAP and increasing doses of V5-RNF166 (1, 2, 3 µg). Cells were treated with 100 µM 6-OHDA for 9 h. Cell lysates were subjected to immunoblotting using anti-Flag or anti-V5 antibody. Relative levels of XIAP are expressed as fold-change relative to the control value obtained from cells transfected with Flag-XIAP alone and treated with 6-OHDA, after normalization against GAPDH (value = 1). Bars represent the mean + SEM from three independent experiments. ***p < 0.001; **p < 0.01; *p < 0.05. **b**–**d** MN9D cells were transiently transfected with the indicated combinations of V5-RNF166 and Flag-XIAP for 48 h. Cells were treated with or without 100 µM 6-OHDA for 9 h. If necessary, cells were further treated with 10 µM MG132 for 4 h prior to preparing lysates. Cell lysates were subjected to immunoprecipitation with anti-Flag M2 affinity gel under denaturing conditions followed by immunoblotting using (**b**) anti-V5 and anti-Flag antibodies or **c** anti-Ub and anti-Flag antibodies. Input was immunoprobed using the indicated antibody. **d** Cell lysates were subjected to immunoblotting using the indicated antibodies. Densitometric values of XIAP and C-cas-3 are expressed as fold-change relative to that of cells transfected with Flag-XIAP alone (C-cas-3) or in combination with V5-RNF166 (XIAP) and treated with 6-OHDA, after normalization against GAPDH (value = 1). Bars represent the mean + SEM from three independent experiments. *p < 0.05. **e** MN9D cells transiently transfected for 48 h with the indicated combinations of siRNF166 RNA and siXIAP RNA were treated with or without 100 µM 6-OHDA for 9 h. Cell lysates were subjected to immunoblotting using anti-cleaved capsase-3 antibody. **f** Rate of cell survival was determined using an MTT reduction assay. Values are expressed as a percentage of the untreated control (100%). Bars represent the mean + SEM of three independent experiments in triplicate. *p < 0.05.

XIAP, a member of the inhibitor of apoptosis (IAP) family, binds to and inhibits caspases via XIAP’s E3 ligase activity–dependent ubiquitination and subsequent proteasome-mediated degradation^34–37^. Having established that RNF166 accelerates caspase activation, we examined whether these events are mediated by RNF166-dependent degradation of XIAP and concomitant caspase activation following 6-OHDA treatment. MN9D cells were transfected with Flag-XIAP and increasing doses of V5-RNF166 and subsequently treated with 6-OHDA. Immunoblotting indicated downregulation of Flag-XIAP after 6-OHDA treatment in MN9D cells transfected with V5-RNF166 (Fig. 7a). Quantitative analysis indicated an apparent inverse correlation between the amount of RNF166 used for transfection and XIAP expression level following 6-OHDA treatment. Co-immunoprecipitation assays indicated enhanced interaction between RNF166 and XIAP after 6-OHDA treatment (Fig. 7b). Cell-based ubiquitination assays indicated that this enhanced interaction upregulated XIAP ubiquitination following 6-OHDA treatment (Fig. 7c; lane 2 vs lane 4). Based on exploratory assays demonstrating RNF166-mediated downregulation of XIAP via ubiquitination, we examined whether these events are involved in the pro-apoptotic mechanism of RNF166. As expected, 6-OHDA–induced caspase-3 cleavage was attenuated in MN9D cells transfected with Flag-XIAP (Fig. 7d; lane 3 vs lane 4). In MN9D cells co-transfected with V5-RNF166 and Flag-XIAP, levels of XIAP protein decreased and cleaved caspase-3 increased after 6-OHDA treatment (Fig. 7d). Consequently, XIAP knockdown increased cleaved caspase-3 levels following 6-OHDA treatment compared with mock-transfected cells (Fig. 7e; lane 1 vs lane 2). Levels of cleaved caspase-3 in RNF166/XIAP double-knockdown cells returned to those of mock-transfected cells (Fig. 7e; lane 1 vs lane 3). MTT reduction assays demonstrated that the rate of enhanced apoptosis in RNF166-knockdown cells returned to that of mock-transfected cells when XIAP was also depleted (Fig. 7f). Collectively, these findings indicate that the pro-apoptotic role of RNF166 involves ubiquitination-dependent degradation of XIAP and consequent caspase activation after 6-OHDA treatment.

## Discussion

6-OHDA is widely used to establish experimental models of PD. To characterize the changes in gene expression following 6-OHDA treatment, we used a TwinChip Mouse-7.4 K microarray with input from MN9D cells. Functional clustering of altered gene sets identified 13 genes encoding proteins with RING domains, among which we focused on RNF166. A previous study reported that RNF125/TRAC-1 exhibits similarity with the functional RING and UIM domains of RNF166, RNF114, and RNF138^38^. They demonstrated abolished E3 ligase activity in RING- and UIM-deficient mutants. Consistent with that report, our present ubiquitination assays demonstrated loss of optimum E3 ligase activity following deletion of the RING or UIM domains of RNF166. Intriguingly, RNF166 was poly-ubiquitinated in the absence of E2 conjugating enzyme (Supplementary Fig. 9). Several E3 ligases that function as unusual chimeric ubiquitin conjugation and ligase (E2/E3) enzymes have been reported^39,40^. Although speculative at present, it is possible that RNF166 exhibits E2/E3 enzyme activity. In situ hybridization and RT-PCR analyses indicated that RNF166 mRNA is widely expressed in the CNS. Immunoblotting demonstrated that the RNF166 protein expression pattern is quite similar to the RNF166 mRNA tissue distribution. Both immunocytochemistry and fractionation immunoblotting demonstrated RNF166 localization in the cytosol and nucleus. Interestingly, we found that RNF166 protein expression in the cerebral cortex is temporally regulated, with maximal expression in the embryonic stage and dramatic downregulation thereafter, with these levels maintained to the adult stage. This suggests that regulation of RNF166 expression is a critical determinant of both development and CNS homeostasis.

**Fig. 8:**
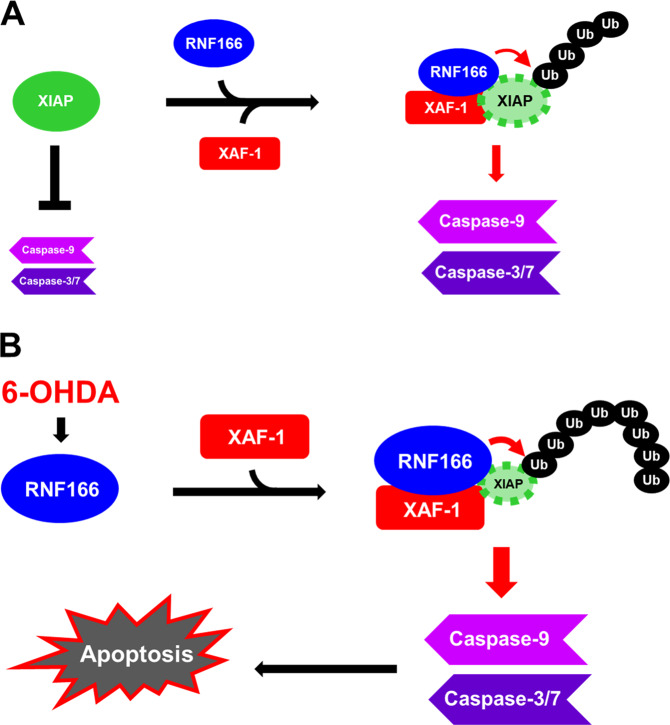
Schematic illustration of a proposed model demonstrating how RNF166 down-regulates XIAP expression and promotes caspase-dependent cell death following 6-OHDA treatment. **a** Under normal conditions, RNF166 interacts with XIAP, and this interaction is enhanced by XAF-1, resulting in a basal level of XIAP polyubiquitination. **b** Under 6-OHDA–induced neuronal death conditions, increased levels of RNF166 interact more vigorously with and degrade XIAP via the ubiquitin-proteasome system, thus inhibiting the pro-survival function of XIAP. Consequently, caspases escape XIAP-mediated degradation, leading to caspase activation and concomitant neuronal apoptosis.

To investigate the pathophysiologic roles of RNF166 in the CNS, we used yeast two-hybrid assay to profile RNF166-interacting proteins (Supplementary Table 1). Among several candidates, the direct interaction between XAF-1 and RNF166 was of particular interest. XAF-1 is a pro-apoptotic tumor suppressor that can bind to and disrupt the anti-caspase activity of XIAP via sequestering XIAP^31,33^. Consequently, XAF-1 antagonizes the anti-caspase activity of XIAP. A recent report by Han et al.^32^ demonstrated that ZNF313 (also known as RNF114) and ZNF228 (a RNF166 homolog) interact with and stabilize XAF-1, ultimately leading to apoptosis in human cancer cells. XAF-1 mediates apoptotic switching of p53 signaling through the activation loop of HIPK2 and ZNF313^41^. The same authors reported that XAF-1 forms a positive feedback loop with interferon regulatory factor-1 (IRF-1) to drive apoptosis via hindering C-terminus of Hsc70-interacting protein–mediated ubiquitination of IRF-1^42^. In our study, we found an interaction of XAF-1 and XIAP with RNF166. All known human IAPs contain one to three copies of the baculovirus IAP repeat (BIR) domain. In addition, several IAPs prevent apoptosis and contain a RING domain, which provides ubiquitin E3 ligase activity^43–45^. As such, several IAPs participate in auto- or trans-ubiquitination and subsequent protein degradation^30,46^. XIAP also contains three BIR domains and N-terminal RING domains. Like many mammalian IAPs, XIAP binds to and inhibits caspase-3, -7, and -9 via different parts of the BIR domain^34,45,47–49^. The E3 ubiquitin ligase activity of the RING domain enables XIAP to catalyze the ubiquitination and subsequent proteasomal degradation of caspases. The in vitro and cell-based assays of our study indicate that RNF166 ubiquitinates XIAP. Consequently, RNF166-mediated degradation of XIAP causes increased caspase activation in 6-OHDA–treated cells. In contrast, RNF166 depletion is linked to decreased caspase-3 activation due to reduced RNF166-mediated XIAP ubiquitination, providing a novel mechanism for the pro-apoptotic role of RNF166 during neurodegeneration. In light of the potential role of XAF-1 in these processes, we demonstrated that RNF166’s interaction with and resulting inhibition of the XIAP anti-caspase activity is further enhanced by XAF-1. Thus, we propose a novel mechanism by which RNF166 in assistance with XAF-1 down-regulates XIAP to promote caspase-dependent apoptosis of 6-OHDA–treated neuronal cells (Fig. 8).

Recent studies demonstrated decreases in the XAF-1 level after treatment with anti-cancer drugs^31–33^. Interestingly, these drugs increased ROS generation in cancer cells; co-treatment with N-acetyl-cysteine (NAC), a generally used antioxidant, reduced ROS generation and XAF-1 expression. In contrast, the XIAP level decreased after treatment with apoptosis-inducing drugs. Co-treatment with anti-oxidants restored XIAP expression. These results suggest that ROS mediate an increase in XAF-1 and decrease in XIAP expression during apoptosis. Our previous studies demonstrated that intracellular ROS levels increase in various neuronal cells following 6-OHDA treatment^10–13^. Furthermore, we demonstrated that 6-OHDA–induced ROS generation leads to caspase activation. To reveal the pro-apoptotic role of RNF166, ROS levels were measured in 6-OHDA–treated/–untreated mock-transfected and RNF166-overexpressing MN9D cells using a cell-permeable ROS-sensitive fluorescent probe, 2′,7′- dichlorodihydrofluorescein diacetate. Levels of dichlorodihydrofluorescein-sensitive ROS increased in MN9D cells overexpressing RNF166, regardless of 6-OHDA treatment (Supplementary Fig. 10A, B). MTT reduction assays indicated RNF166-mediated acceleration of 6-OHDA– and H_2_O_2_-induced cell death (Supplementary Fig. 10C), indicating that RNF166 promotes apoptosis of drug–treated MN9D cells by increasing intracellular ROS levels. NAC significantly inhibited cell death induced by 6-OHDA (Supplementary Fig. 10D). The rate of inhibition was comparable to that of mock-transfected MN9D cells co-treated with NAC, suggesting that RNF166-mediated upregulation of apoptosis is due at least in part to its ROS-inducing activity. In support of this notion, we found that RNF166-mediated acceleration of cell death is detected in cells treated with lower doses (0.5 mM and 1 mM) but not with higher doses of hydrogen peroxide (above 1.5 mM). It would be interesting to determine how RNF166-induced ROS generation activates ROS-dependent signaling pathways and further mediates neuronal cell death. Similarly, it is worth investigating whether RNF166-induced ROS generation regulates XAF-1 and XIAP expression.

In summary, our present study identified an E3 ligase gene, RNF166, as a novel pro-apoptotic protein in both naturally occurring and ROS-induced neuronal death. The E3 ubiquitin ligase activity of RNF166 is particularly important in regulating 6-OHDA–induced apoptosis via degradation of XIAP and subsequent enhanced activation of caspase. In terms of functionality, we proposed a scenario in which RNF166 exerts its pro-apoptotic role by initially inducing ROS generation and terminally activating caspases. Our recent LC-MS/MS analysis revealed several other biding proteins of RNF166 which are involved in the process of DNA damage and repair (data not shown). In consideration of a ubiquitous expression of RNF166 in the brain, we have speculated that RNF166 may play a critical role in a wide range of pathophysiological processes in the brain. Further studies using other RNF166 binding partners are needed to elucidate its role in the CNS. In consideration of its expression in the cytosol and the nucleus, similarly, role of RNF166 present in the distinct subcellular compartments remains to be determined.

## Materials and methods

### Microarray data display

Previously, we performed a microarray analysis (TwinChip Mouse-7.4 K; Digital Genomics, Seoul, Korea) using mRNA harvested from 6-OHDA-treated MN9D dopaminergic neuronal cells at various time points^27^. The primary data were clustered using keywords including RING domain (*q*-value ≤ 0.05). The data were represented graphically by coloring each cell, based on the measured fluorescence ratio using TreeView, version 1.60 (http://rana.lbl.gov/EisenSoftware.htm)^28^. Cells exhibiting a log ratio of zero (virtually no change) were colored in black. Increasingly positive and negative log ratios were accordingly marked with increasing intensity of red and green, respectively.

### RNA isolation from MN9D cells and mouse tissues

Total RNA was harvested using Trizol reagent (Invitrogen, San Diego, CA, USA) from MN9D cells treated with 6-OHDA for the indicated time periods). Tissues were dissected from 2-week-old crl:CD-1 male mice (Orientbio Seongnam-Si, Korea) in accordance with the protocol of Jackson-Lewis and Przedborski^50^. Total RNA was isolated from the indicated brain regions as described above. All animal experiments were conducted in accordance with the guidelines set by Yonsei University (2018-01-689-01). No blinding was done when brain tissues were subjected to analyses.

### RT-PCR and qPCR

RT-PCR was performed to analyze RNF166 mRNA expression levels and patterns. Briefly, purified RNA (5 μg) from 6-OHDA–treated MN9D cells or mouse tissues was mixed with M-MLV reverse transcriptase and 0.5 μg of random primer (all from Promega, Madison, WI, USA). In the PCR reaction, 5 μL of cDNA was mixed with mRNF166-specific primers: forward, 5′-ACAAGGCCACCCATGTAGAGAA-3′; reverse, 5′-ATACCACACGGTTGGGA TCACT-3′. Universal 18S primers (Ambion Inc., Austin, TX, USA) were included in the reaction mixes as expression controls. For qPCR, total RNA from non-treated MN9D cells or cells treated with 6-OHDA for various time periods was incubated with DNAse I (Promega) for 1 h at 37 °C then reverse transcribed using Topscrit RT DryMix(dT18) (Enzynomics, Seoul, Korea) according to the manufacturer’s instructions. All samples within an experiment were reverse transcribed at the same time, and the resulting cDNA was diluted 1:4 in nuclease-free water. An ABI 7300 sequence detection system (Applied Biosystems, Foster City, CA, USA) was used for qPCR, and amplifications were carried out using SYBR Premix Ex Taq II (TaKaRa) and the following primers: mouse RNF166 forward 5′-ACCTGCCAAGTATGATGACATCA-3′, reverse 5′-GGTCCTCAGTGTAGCCCAAGAT-3′; mouse GAPDH forward 5′-CAATGACCCCTTCATTGACC-3′, reverse 5′-GACAAGCTTCCCGTTCTCAG-3′. Relative gene expression was quantified using the Pfaffl method^51^, with the fold-change in gene expression normalized to that of an internal control gene (GAPDH).

### In situ hybridization

PCR fragments encompassing nucleotides 1–500 (probe #1), 122–421 (probe #2), or 200–400 (probe #3) of mouse RNF166 were subcloned into the p-GEM T easy vector (Promega). The PCR primer sequences used to generate each probe were as follows: probe #1 (1–500): forward 5′-ATGGCGATGTTCCGCAGCC-3′ and reverse 5′- TTCACCAGCTCCTGCTGATCCA-3′; probe #2 (122–421): forward 5′-ATCGGCCAGTGGCCATCG-3′ and reverse 5′-GGATGGGCTGAGATGTGGGC-3′; probe #3 (200–400): forward 5′-CTCTGTGCCCACTCTGCCGTC-3′ and reverse 5′-CCACAGGGACGAATT TGGGG-3′. In situ hybridization was performed as previously described, with some modifications^52^. Vectors containing RNF166 sequences were transcribed with T3 RNA polymerase to obtain antisense riboprobes. Linearized DNA was labeled with [^35^S]-UTP (Amersham, Cardiff, UK) by in vitro transcription. A 1-μL aliquot of labeled mRNA was suspended in 4 mL of scintillation fluid, and activity (in cpm) was determined using a scintillation counter. Subsequently, tissue sections were permeabilized with proteinase K (1 μg/mL, 37 °C, 30 min), treated with acetic anhydride in 0.1 M triethanolamine (pH 8.0), washed in 2× saline–sodium citrate (SSC) (pH 7.0), and transferred into 500 μL of hybridization solution in 24-well culture plates. The hybridization solution consisted of 50% formamide, 0.01% polyvinyl pyrrolidone, 0.01% Ficoll, 0.01% bovine serum albumin, 50 μg/mL denatured salmon sperm DNA, 250 μg/mL yeast tRNA, 40 mM dithiothreitol (DTT), 10% dextran sulfate, and [^35^S]-labeled RNF166 cRNA probes at 1 × 10^7^ cpm/mL. Sections were hybridized with the RNF166 riboprobe for 18 h at 55 °C in hybridization solution. After overnight incubation in a humidified chamber, sections were washed twice for 30 min at 55 °C in 4× SSC containing 5 mM DTT, treated with RNase A (20 μg/mL) for 30 min at 45 °C, and washed four times (15 min/wash) in 2× SSC containing 5 mM DTT at room temperature. The sections were then washed twice (30 min/wash) in 0.5× SSC containing 5 mM DTT, once for 30 min in 0.1× SSC containing 5 mM DTT at 50–55 °C, and once in 0.1× SSC with 5 mM DTT at room temperature. Sections were then mounted on gelatin-coated microscope slides, air-dried overnight, and then exposed to Hyperfilm β-max (Amersham) for 3 days.

### Cell culture and drug treatment

MN9D dopaminergic neuronal cells were established via somatic fusion between embryonic mesencephalic neurons and N18TG cells^53^. MN9D cells were plated in 24-well or P-100 culture plates (Corning Glass Works, Corning, NY, USA) coated with 25 μg/mL poly-d-lysine (Sigma). Cultures were maintained in Dulbecco’s modified Eagle’s medium (DMEM; Sigma) supplemented with 10% fetal bovine serum (FBS; Gibco, Grand Island, NY, USA) in an incubator with an atmosphere of 10% CO_2_ at 37 °C for 3 days. The culture medium was then switched to serum-free N2. HEK293 cells, originally purchased from the American Type Culture Collection, were cultured in DMEM (GenDEPOT, Barker, TX, USA) supplemented with 10% heat-inactivated FBS (GenDEPOT) at 37 °C in an atmosphere of 5% CO_2_ for 2–3 days. If necessary, cells were treated as follows: 100 μM 6-OHDA (Sigma), 2.5 mM lactacystin (AG Scientific, San Diego, CA, USA), 10 µM MG132 (Enzo Life Sciences Inc., Farmingdale, NY, USA), or 1 mM NAC (Sigma). Concentrations and durations of drug treatment were empirically determined and previously reported by us^10–12,54–56^.

### Primary hippocampal neuron culture

As described previously^54^, primary hippocampal neuron cultures were prepared by removing the hippocampus from gestation day 17 Sprague-Dawley rat embryos (Orientbio). Briefly, mechanically and enzymatically dissociated cells were plated at 1 × 10^5^ cells per 1.4 cm^2^ Aclar embedding film (Electron Microscopy Sciences, Fort Washington, PA, USA) pre-coated with 100 μg/mL poly-d-lysine (Sigma) and 4 μg/mL laminin (Invitrogen). Hippocampal neurons were maintained at 37 °C in a humidified 5% CO_2_ atmosphere in MEM (Gibco) supplemented with 10% FBS, 2 mM glutamine, 1 mM sodium pyruvate, 10 units penicillin-streptomycin, and 6.0 g/mL glucose (all from Sigma). Twenty-four-hours after plating, the medium was changed to Neurobasal medium (Gibco) supplemented with 2% B27 (Invitrogen), 1 mM glutamine, and 6.0 g/mL glucose. After changing to fresh medium at days in vitro (DIV) 3, 5 μg/mL cytosine β-D-arabinofuranoside (Sigma) was added to the cultures. All animal experiments were conducted in accordance with the guidelines for animal care and used set and approved by Yonsei University (2018-01-689-01).

### Transfection and lentiviral infection

Overexpression and knockdown experiments were carried out basically as previously described^54,55^. Briefly, MN9D cells (1.0 × 10^6^) were plated on P-100 culture plates. After 2 days, cells were transfected with predetermined amounts of plasmids using Lipofectamine 2000 as recommended by the supplier (Invitrogen). For transient transfection, 1.5 × 10^6^ HEK293 cells were plated on P-100 culture plates. After 3 days, cells were transfected with predetermined amounts of plasmids using polyethylenimine (Sigma). Hippocampal neurons at DIV1 were transiently transfected with plasmids using Lipotectamine 2000. For knockdown experiments, cells were transiently transfected with either control nontarget siRNA (Genolution, Seoul, Korea) or MISSION siRNA directed against RNF166 (Genolution; #1) or XIAP (Bioneer, Daejeon, Korea; #1). RNF166 siRNA (siRNA/RNF166) was synthesized as follows: sense 5′-AUCUUAUCCUGUGUGAUAAUU-3′; control siRNA was synthesized as follows: sense 5′- CCUCGUGCCGUUCCAUCAGGUAGUU-3′. XIAP siRNA was synthesized as follows: sense 5′-CAGAACACAGGAGACACUU-3′. All siRNA transfections used Lipofectamine 2000. Stable knockdown experiments used two pLKO.1-purolentiviral vectors (Sigma) encoding different shRNA sequences targeting the transcript of murine RNF166 (shRNA #1, 5′-CCAAGATGAGAGCGCACATTT-3′; shRNA #3, 5′-TGGTGAAGCACTGCGTGGAAA-3′) and blank shRNA negative control. HEK293 cells (1 × 10^6^) were transfected using 15 μg of RNF166 or blank vectors, 5 μg of vesicular stomatitis virus envelope G glycoprotein, VSVg plasmid, and 10 µg of packaging construct, ΔNRF plasmid. VSVg and ΔNRF plasmid were kindly provided by Dr. Mandel (Haifa, Israel). At 24 to 48 h post-transfection, lentiviruses were harvested and concentrated using a 0.45-µm filter (Millipore, Billerica, MA, USA). Upon reaching approximately 70–80% confluence, MN9D cells were infected twice with lentiviral particles. Each round of infection was carried out for 12 h in the presence of 8 µg/mL polybrene. Forty-eight hours after infection, stable MN9D cells were selected in the presence of puromycin (5 μg/mL).

### Plasmids

The human RNF166 gene (hRNF166; accession number; NM_178841) was subcloned into the *Bam*HI and *Xho*I sites of the pcDNA 3.1-V5 His vector (Invitrogen) from Ultimate ORF Clones (clone ID; IOH14416; Invitrogen). The primers used were: forward: 5′-ATGGATCCATGGCTATGTTCCGCAGC-3′, reverse: 5′-CGCTCGAGGTTCTCAGAGAGAGACAGGG-3′. hRNF166 deletion mutants encoding RNF166 ΔC (1–199) and ΔN (110–237) were generate by PCR using pcDNA3.1-V5/hRNF166 as a template and inserted into the same vector. Double point mutant cDNA encoding RNF166 C33, 36S was generated via site-directed mutagenesis, generally as recommended by the manufacturer (Prime START HS DNA polymerase; TaKaRa Bio Inc, Otsu, Japan). The mouse RNF166 gene (mRNF166; accession number; NM_001033142) was generated by RT-PCR using available templates in the crl:CD-1 male mouse whole brain. Amplified mRNF166 cDNA was subcloned into the *Bam*HI and *Xho*I sites of the pcDNA 3.1-V5 His vector. The primers used were: forward: 5′-TAGGATCCATGGCGGGCTTGGG-3′, reverse: 5′-CGCTCGAGGTTCTCAGAGAGGGACAGAG-3′. The mRNF166 gene was subcloned into the *Eco*R I and *Xba*I sites of modified pCI-neo-V5, pCI-neo-FLAG, or pCI-neo-HA plasmids (Promega). The primers used were: Primer 1: 5′-GGAATTCCATGGCGATGTTCCGCAGCC-3′, Primer 2: 5′-GCTCTAGATCAGTTCTCAGAGAGGGAC-3′. N-terminal Flag-tagged mRNF166 was generated by PCR and subcloned into the *Eco*RI and *Sal*I sites of the p3X FLAG-CMV-7.1 vector (Sigma). mRNF166 with GFP-tag at either the N- or C-terminus was generated by PCR and subcloned into the *Hind*III and *Sal*I sites of the pAcGFP-N1 or -C1 vector, respectively (Clontech Laboratories, Inc., Mountain View, CA, USA). Flag-tagged wild-type human XIAP and ΔRING constructs were generously provided by Dr. T.H. Lee of Yonsei University (Seoul, Korea). Flag-tagged human XIAP H467A was generated via site-directed mutagenesis. The primers used were: Primer 1: 5′-CATTGTTTACAAGTGACTAGAGCTCCACAAGGAACAAAAACGATAGC-3′, Primer 2: 5′-GCTATCGTTTTTGTTCCTTGTGGAGCTCTAGTCACTTGTAAACAATG-3′. The human XAF-1 gene was subcloned into the *Eco*RI and *Sal*I sites of the modified pCI-neo-Flag or pCI-neo-V5 plasmids (Promega). pEF/HA-ubiquitin vector was kindly provided by Dr. T.H. Lee of Yonsei University. HA-tagged human ubiquitin was provided by Dr. K.C. Chung of Yonsei University and used in a previous study^56^.

### Immunoprecipitation and denaturing immunoprecipitation

MN9D and HEK293 cells were washed with ice-cold PBS containing 1 mM EDTA and lysed in RIPA lysis buffer. Cell lysates were centrifuged at 13,000 × *g* for 10 min at 4 °C. Proteins in the supernatants were quantified using Bradford protein assay reagent (Bio-Rad, Hercules, CA, USA). After pre-clearing cell lysates with protein A-agarose (Upstate Biotechnology, Lake Placid, NY, USA) for 30 min at 4 °C, protein (0.5 mg) from each sample was incubated overnight at 4 °C with 0.5 mg of indicated antibody or 10 μL of anti-FLAG M2 affinity gel (Sigma) with gentle rotation. The samples (except the sample containing anti-FLAG M2 affinity gel) were incubated with protein A-agarose for 2 h at 4 °C and washed five times with 1 mL of RIPA buffer (50 mM Tris-HCl [pH 7.4], 150 mM NaCl, 1% NP-40, 0.25% sodium deoxycholate, 1 mM EGTA) by centrifugation at 3000 × *g* for 2 min at 4 °C. Proteins bound to the agarose beads were eluted by adding 30 μL of 1× sample buffer and denatured by boiling for 5 min. For denaturing immunoprecipitation using MN9D cells, the first round was performed as described above. Following three washes with RIPA buffer, immunoprecipitated samples were denatured using 1% SDS RIPA buffer and then boiled for 5 min. Denatured samples were diluted with 10 volumes of RIPA buffer without SDS and then subjected to the second round of immunoprecipitation. The bound proteins were eluted from the beads by addition of 30 μL of 1× protein sample buffer, denatured by boiling for 5 min, and subjected to immunoblotting using the antibodies indicated above.

### Immunoblotting

Cell and tissue lysates were prepared by solubilization in cold RIPA buffer containing a protease inhibitor cocktail (Roche, Mannheim, Germany). Lysates were centrifuged at 13,000 *g* at 4 °C for 15 min, and the protein concentrations were determined using the bicinchoninic acid protein assay (Bio-Rad). Between 30 and 70 μg of protein was separated on a predetermined percentage SDS-PAGE gel and blotted onto PVDF membranes (Pall, Ann Arbor, MI, USA). The blotted membranes were then probed with one of the following primary antibodies: anti-RNF166 (1:1000; Sigma), anti-ub (1:1000; Santa Cruz Biotechnology, Dallas, TX, USA), anti-XIAP (1:1000; Cell Signaling, Beverly, MA, USA), anti-cleaved caspase-3 (1:2000; Cell Signaling), anti-cleaved caspase-9 (1:2000; Cell Signaling), anti-cleaved caspase-2 (1:2000; Abcam, Cambridge, UK), anti-actin (1:3000; Sigma), anti-GAPDH (1:3000; Santa Cruz Biotechnology, Santa Cruz, CA, USA), anti-GST (1:3000; Santa Cruz Biotechnology), anti-HA-peroxidase (3F10, 1:3000; Roche), anti-Flag-HRP (1:5000; Sigma), or anti-V5-HRP (1:5000; Invitrogen). For fraction markers, anti-superoxide dismutase 1 (1:1000, Santa Cruz Biotechnology) and anti-laminA/C (1:1000; Santa Cruz Biotechnology) were used. Specific bands were detected by enhanced chemiluminescence (Amersham Pharmacia Biotech, Piscataway, NJ, USA).

### Immunocytochemistry

MN9D cells cultured on 100 μg/mL poly-d-lysine-coated aclar film (Electron Microscopy Science, Washington, PA, USA) were placed in a 4-well culture plate (Nunc, Roskilde, Denmark) and transiently transfected with the indicated plasmids. Twenty-four-hours post-transfection, cells were washed with PBS and fixed with either 4% paraformaldehyde at room temperature or 100% methanol at −20 °C for 20 min. Primary hippocampal neurons were fixed with 4% paraformaldehyde at room temperature for 20 min. The cells were then blocked for 1 h in PBS containing 5% normal goat serum and 0.1% Triton X-100, followed by incubation at 4 °C overnight with anti-V5 (1:200; Invitogen), anti-FLAG (1:200), or anti-NeuN (1:200, Millipore) antibodies in PBS containing 1% normal goat serum and 0.1% Triton X-100. After several washes with PBS, fluorescent secondary antibodies were used: Alexa 568-conjugated goat anti-mouse IgG (1:200; Molecular Probes, Eugene, OR, USA) or Alexa 488-conjugated goat anti-rabbit IgG (1:200; Molecular Probes). Nuclei were counterstained with 1 mg/mL Hoechst 33258 (Molecular Probes), and cells were examined under an LSM 700 META confocal microscope equipped with epifluorescence and an LSM digital image analyzer (Carl Zeiss, Zena, Germany). For microscopic examinations, we were blinded to the group allocation during experiments, data collection and assessing outcome.

### TUNEL assays and annexin V staining

For TUNEL assays, GFP-tagged RNF166-transfected primary hippocampal cells were fixed with 4% paraformaldehyde at room temperature for 20 min, incubated in permeabilization solution (0.1% Triton X-100 in 0.1% sodium citrate) for 2 min on ice, and then washed twice with PBS. The cells were stained with TUNEL reaction mixture (In situ Cell Death Detection Kit, TMR red, Roche) for 1 h at room temperature in a humidified atmosphere in the dark. After washing twice with PBS, cells on the aclar film were mounted on slide glass and examined under a Radiance 2100 laser-scanning confocal microscope (Bio-Rad). As long as more than one GFP-positive signal was detected per micrograph, 40 randomly selected micrographs were counted for each sample. To assess apoptotic features, MN9D cells were subjected to annexin V staining using an FITC-conjugated Annexin V Apoptosis Detection Kit I (BD Bioscience, San Diego, CA, USA). The cells were then analyzed on a FACSCalibur instrument (BD Biosciences, Mountain View, CA) according to the manufacturer’s instructions.

### In vitro and cell-based ubiquitination assays

For in vitro ubiquitination assays, the reaction mixture included 1–2 μg of GST or GST-RNF166, 300 ng of E1 (UBE1; Boston Biochem, MA, USA), 300 ng of E2 (Boston Biochem), 1 μg of ubiquitin (Sigma), 0.01% glycerol, and 2 mM ATP in buffer consisting of 50 mM Tris-HCl (pH 7.5), 2.5 mM MgCl_2_, 15 mM KCl, and 0.7 mM DTT. The final reaction volume was 10 μL. After 45 min of incubation at 37 °C, conjugation reactions were quenched by adding 5× protein sample buffer. An equal amount of each sample was subjected to SDS-PAGE. Cell-based ubiquitination assays were performed basically as described previously^57^. Briefly, MN9D cells were transiently transfected with the combination of indicated plasmids. At 24 h post-transfection, cells were lysed with 1% Triton X-100 in PBS. Cell lysates were pre-cleared of bead-binding proteins by incubation with protein A-agarose (Upstate, Lake Placid, NY, USA). Anti-V5 antibody (Invitrogen; 1 mg) was added to pre-cleared cell lysates and incubated at 4 °C overnight. The beads were incubated for 1 h at 4 °C, washed, and then heated at 98 °C for 5 min in RIPA buffer containing 1% SDS. Beads diluted with RIPA buffer (1:10) were re-immunoprecipitated with anti-V5 antibody and beads. Samples were then subjected to immunoblotting.

### GST pull-down assay

To prepare recombinant GST-fused proteins, *E. coli* BL21 was transformed with the pGEX4T-1 vector encoding the mouse RNF166 sequence. Transformed *E. coli* cells were incubated in 0.1 mM isopropyl β-D-1-thiogalactopyranoside for 15 h at 30 °C. Pelleted bacteria were resuspended in *E. coli* lysis buffer (30 mM Tris-HCl [pH 7.5], 0.1 mM NaCl, 1 mM DTT, 1% Triton X-100, protease inhibitor cocktail [Roche]), sonicated on ice, and centrifuged at 3,000 × *g* for 15 min at 4 °C. The resulting supernatants were incubated with glutathione Sepharose 4D beads overnight at 4 °C, after which the beads were washed five times with cold PBS and eluted with buffer consisting of 20 mM reduced glutathione in 50 mM Tris-HCl (pH 8.0). All reagents were purchased from Sigma unless stated otherwise. After the elution step, the buffer was exchanged with GST binding buffer (25 mM Tris-HCl [pH 7.5], 1 mM DTT, 30 mM MgCl_2_, 40 mM NaCl, and 0.5% NP40). The eluents were resolved by SDS-PAGE and stained with 0.1% Coomassie brilliant blue G-250 (Amresco, Solon, OH, USA). For pull-down assays, 30 μg of GST-fused proteins was incubated for 1 h at 4 °C with 200 μL of bead-containing binding buffer. Cell lysate (2 mg) of Flag-tagged human XIAP- or Flag-tagged human XAF-1–overexpressing HEK293 cells was added to binding buffer containing GST-fused proteins, and the mixtures were incubated overnight at 4 °C. Following incubation, the beads were washed five times with wash buffer I (25 mM Tris-HCl [pH 7.5], 1 mM DTT, 30 mM MgCl_2_, 40 mM NaCl, 1% NP40) and washed three times with wash buffer II (25 mM Tris-HCl [pH 7.5], 1 mM DTT, 30 mM MgCl_2_, 40 mM NaCl). After washing, 35 μL of 1× sample buffer was added to 25 μL of the samples and subjected to SDS-PAGE followed by immunoblotting.

### Cell viability assay

Following incubation with various experimental reagents, the rate of cell survival was assessed via colorimetric measurement of MTT reduction^58^. In brief, a final concentration of 1 mg/mL MTT solution was added to cell cultures following treatment with the indicated drugs for predetermined time periods. The cells were then incubated for 1 h at 37 °C and then lysed with 20% SDS in 50% aqueous dimethylformamide for 24 h. The optical density of the dissolved formazan grains was measured at 540 nm using a microplate reader (Molecular Devices, Palo Alto, CA, USA). Values from triplicate wells were calculated as a percentage relative to the untreated control (defined as 100% survival).

### DNA fragmentation assays

To assess DNA fragmentation, MN9D cells were treated with 100 μM 6-OHDA for 18 h. Soluble DNA was harvested by lysing cells for 30 min at 50 °C in buffer consisting of 0.5% Triton X-100, 5 mM Tris-HCl (pH 7.4), 20 mM EDTA, and 20 μg/mL proteinase K. Following microcentrifugation at 13,000 *g* at 4 °C for 15 min, DNA in the supernatant was extracted with phenol/chloroform and ethanol precipitated. DNA precipitates were dissolved in an appropriate amount of double-distilled H_2_O and incubated with 4 mg/mL RNase A (Promega) for 1 h at 37 °C. Purified DNA (30 μg) was electrophoresed on 1.5% agarose gels and stained with ethidium bromide. Intercalated DNA bands were viewed on a UV transilluminator using a Molecular Imager Gel Dox XR system (Bio-Rad).

### Measurement of intracellular ROS levels

To measure intracellular ROS levels, MN9D cells treated with 100 μM 6-OHDA for 9 h were first dissociated with 0.25% trypsin. Singly dispersed cells were stained with 3 μM 5-(and-6)-chloromethyl-2′ 7′-dichlorodihydrofluorescein diacetate acetyl ester (Molecular Probes) and dihydroethidium (Molecular Probes) in DMEM for 30 min at 37 °C in an incubator with an atmosphere of 10% CO_2_. Cells were washed twice with PBS. Intracellular levels of fluorescent dye–sensitive ROS were determined using a FACSCalibur (BD Biosciences) and calculated as the mean fluorescence intensity using CellQuest software (BD Biosciences).

### Statistical analyses

Sample sizes were chosen based on the similar experiments previously published from which we performed statistical analyses. No data were excluded. Data are expressed as the mean ± SEM from the indicated number of experiments. The statistical significance of differences between two samples was analyzed using the Student’s *t*-test, and differences between multiple samples by analysis of variance with post hoc Tukey’s multiple comparison test using GraphPad Prism 5. Values of ****p < 0.0001, ***p < 0.001, **p < 0.01 or *p < 0.05 were considered significant. ‘ns’ indicates not significant.

## Supplementary information

Supplementary Table 1

Supplementary Figure 1

Supplementary Figure 2

Supplementary Figure 3

Supplementary Figure 4

Supplementary Figure 5

Supplementary Figure 6

Supplementary Figure 7

Supplementary Figure 8

Supplementary Figure 9

Supplementary Figure 10

## References

[1] Goedert M, Spillantini MG, Del Tredici K, Braak H (2013). 100 years of Lewy pathology. Nat. Rev. Neurol..

[2] Martin I, Dawson VL, Dawson TM (2011). Recent advances in the genetics of Parkinson’s disease. Annu. Rev. Genomics Hum. Genet..

[3] Dexter DT (1994). Indices of oxidative stress and mitochondrial function in individuals with incidental Lewy body disease. Ann. Neurol..

[4] Hirsch EC, Jenner P, Przedborski S (2013). Pathogenesis of Parkinson’s disease. Mov. Disord..

[5] Schapira AH (2007). Mitochondrial dysfunction in Parkinson’s disease. Cell Death Differ..

[6] Ray PD, Huang BW, Tsuji Y (2012). Reactive oxygen species (ROS) homeostasis and redox regulation in cellular signaling. Cell. Signal..

[7] Dexter DT (1989). Basal lipid peroxidation in substantia nigra is increased in Parkinson’s disease. J. Neurochem..

[8] Jenner P (1996). Oxidative stress in Parkinson’s disease and other neurodegenerative disorders. Pathol. Biol..

[9] Porter CC, Totaro JA, Burcin A (1965). The relationship between radioactivity and norepinephrine concentrations in the brains and hearts of mice following administration of labeled methyldopa or 6-hydroxydopamine. J. Pharmacol. Exp. Ther..

[10] Choi WS (1999). Two distinct mechanisms are involved in 6-hydroxydopamine- and MPP+-induced dopaminergic neuronal cell death: role of caspases, ROS, and JNK. J. Neurosci. Res..

[11] Han BS (2003). Caspase-dependent and -independent cell death pathways in primary cultures of mesencephalic dopaminergic neurons after neurotoxin treatment. J. Neurosci..

[12] Choi WS (2004). Phosphorylation of p38 MAPK induced by oxidative stress is linked to activation of both caspase-8- and -9-mediated apoptotic pathways in dopaminergic neurons. J. Biol. Chem..

[13] Lee YM (2008). Oxidative modification of peroxiredoxin is associated with drug-induced apoptotic signaling in experimental models of Parkinson disease. J. Biol. Chem..

[14] DeMartino GN, Slaughter CA (1999). The proteasome, a novel protease regulated by multiple mechanisms. J. Biol. Chem..

[15] Gómez-Díaz C, Ikeda F (2019). Roles of ubiquitin in autophagy and cell death. Semin. Cell Dev. Biol..

[16] Betarbet R, Sherer TB, Greenamyre JT (2005). Ubiquitin-proteasome system and Parkinson’s diseases. Exp. Neurol..

[17] Ciechanover A, Brundin P (2003). The ubiquitin proteasome system in neurodegenerative diseases: sometimes the chicken, sometimes the egg. Neuron.

[18] Deshaies RJ, Joazeiro CA (2009). RING domain E3 ubiquitin ligases. Annu. Rev. Biochem..

[19] Miranda M, Sorkin A (2007). Regulation of receptors and transporters by ubiquitination: new insights into surprisingly similar mechanisms. Mol. Interv..

[20] Berndsen CE, Wolberger C (2014). New insights into ubiquitin E3 ligase mechanism. Nat. Struct. Mol. Biol..

[21] Cohen P, Tcherpakov M (2010). Will the ubiquitin system furnish as many drug targets as protein kinases. Cell.

[22] Staropoli JF (2003). Parkin is a component of an SCF-like ubiquitin ligase complex and protects postmitotic neurons from kainate excitotoxicity. Neuron.

[23] Hampe C, Ardila-Osorio H, Fournier M, Brice A, Corti O (2006). Biochemical analysis of Parkinson’s disease-causing variants of Parkin, an E3 ubiquitin-protein ligase with monoubiquitylation capacity. Hum. Mol. Genet..

[24] Matsuda N (2006). Diverse effects of pathogenic mutations of Parkin that catalyze multiple monoubiquitylation in vitro. J. Biol. Chem..

[25] Chen HW (2015). Ring finger protein 166 potentiates RNA virus-induced interferon-β production via enhancing the ubiquitination of TRAF3 and TRAF6. Sci. Rep..

[26] Heath RJ (2016). RNF166 determines recruitment of adaptor proteins during antibacterial autophagy. Cell Rep..

[27] Park B (2011). Microarray expression profiling in 6-hydroxydopamine-induced dopaminergic neuronal cell death. J. Neural Transm..

[28] Eisen MB, Spellman PT, Brown PO, Botstein D (1998). Cluster analysis and display of genome-wide expression patterns. Proc. Natl Acad. Sci. USA.

[29] Varfolomeev E (2007). IAP antagonists induce autoubiquitination of c-IAPs, NF-kappaB activation, and TNFalpha-dependent apoptosis. Cell.

[30] Yang Y, Fang S, Jensen JP, Weissman AM, Ashwell JD (2000). Ubiquitin protein ligase activity of IAPs and their degradation in proteasomes in response to apoptotic stimuli. Science.

[31] Fong WG (2000). Expression and genetic analysis of XIAP-associated factor 1 (XAF1) in cancer cell lines. Genomics.

[32] Han J (2013). ZNF313 is a novel cell cycle activator with an E3 ligase activity inhibiting cellular senescence by destabilizing p21(WAF1.). Cell Death Differ..

[33] Liston P (2001). Identification of XAF1 as an antagonist of XIAP anti-caspase activity. Nat. Cell Biol..

[34] Deveraux QL, Takahashi R, Salvesen GS, Reed JC (1997). X-linked IAP is a direct inhibitor of cell-death proteases. Nature.

[35] Huang Y (2001). Structural basis of caspase inhibition by XIAP: differential roles of the linker versus the BIR domain. Cell.

[36] Suzuki Y, Nakabayashi Y, Takahashi R (2001). Ubiquitin-protein ligase activity of X-linked inhibitor of apoptosis protein promotes proteasomal degradation of caspase-3 and enhances its anti-apoptotic effect in Fas-induced cell death. Proc. Natl Acad. Sci. USA.

[37] Silke J (2002). The anti-apoptotic activity of XIAP is retained upon mutation of both the caspase 3- and caspase 9-interacting sites. J. Cell Biol..

[38] Giannini AL, Gao Y, Bijlmakers MJ (2008). T-cell regulator RNF125/TRAC-1 belongs to a novel family of ubiquitin ligases with zinc fingers and a ubiquitin-binding domain. Biochem. J..

[39] Bartke T, Pohl C, Pyrowolakis G, Jentsch S (2004). Dual role of BRUCE as an antiapoptotic IAP and a chimeric E2/E3 ubiquitin ligase. Mol. Cell.

[40] Chang TL, Cubillos FF, Kakhniashvili DG, Goodman SR (2004). Ankyrin is a target of spectrin’s E2/E3 ubiquitin-conjugating/ligating activity. Cell. Mol. Biol..

[41] Lee MG (2014). XAF1 directs apoptotic switch of p53 signaling through activation of HIPK2 and ZNF313. Proc. Natl Acad. Sci. USA.

[42] Jeong SI (2018). XAF1 forms a positive feedback loop with IRF-1 to drive apoptotic stress response and suppress tumorigenesis. Cell Death Dis..

[43] Birnbaum MJ, Clem RJ, Miller LK (1994). An apoptosis-inhibiting gene from a nuclear polyhedrosis virus encoding a polypeptide with Cys/His sequence motifs. J. Virol..

[44] Crook NE, Clem RJ, Miller LK (1993). An apoptosis-inhibiting baculovirus gene with a zinc finger-like motif. J. Virol..

[45] Srinivasula SM, Ashwell JD (2008). IAPs: what’s in a name. Mol. Cell.

[46] Hanson AJ (2012). XIAP monoubiquitylates Groucho/TLE to promote canonical Wnt signaling. Mol. Cell.

[47] Deveraux QL, Reed JC (1999). IAP family proteins–suppressors of apoptosis. Genes Dev..

[48] Duckett CS (1998). Human IAP-like protein regulates programmed cell death downstream of Bcl-xL and cytochrome c. Mol. Cell. Biol..

[49] Shiozaki EN (2003). Mechanism of XIAP-mediated inhibition of caspase-9. Mol. Cell.

[50] Jackson-Lewis V, Przedborski S (2007). Protocol for the MPTP mouse model of Parkinson’s disease. Nat. Protoc..

[51] Pfaffl MW (2001). A new mathematical model for relative quantification in real-time RT-PCR. Nucleic Acids Res..

[52] Park SH (2008). Effects of repeated citalopram treatments on chronic mild stress-induced growth associated protein-43 mRNA expression in rat hippocampus. Korean. J. Physiol. Pharmacol..

[53] Choi HK, Won L, Roback JD, Wainer BH, Heller A (1992). Specific modulation of dopamine expression in neuronal hybrid cells by primary cells from different brain regions. Proc. Natl Acad. Sci. USA.

[54] Lee J (2018). The acetylation of cyclin-dependent kinase 5 at lysine 33 regulates kinase activity and neurite length in hippocampal neurons. Sci. Rep..

[55] Kim C (2016). Phosphorylation of CHIP at Ser20 by Cdk5 promotes tAIF-mediated neuronal death. Cell Death Differ..

[56] Hwang JY (2016). Proteolytic degradation and potential role of onconeural protein cdr2 in neurodegeneration. Cell Death Dis..

[57] Nagai H (2009). Ubiquitin-like sequence in ASK1 plays critical roles in the recognition and stabilization by USP9X and oxidative stress-induced cell death. Mol. Cell.

[58] Hansen MB, Nielsen SE, Berg K (1989). Re-examination and further development of a precise and rapid dye method for measuring cell growth/cell kill. J. Immunol. Methods.

