# Supplementary figures and images for: RING-finger protein 166 plays a novel pro-apoptotic role in neurotoxin-induced neurodegeneration via ubiquitination of XIAP
Source: Cell Death Dis. 2020 Oct 31;11(10):939. doi: 10.1038/s41419-020-03145-x (PMC7603511; doi:10.1038/s41419-020-03145-x)

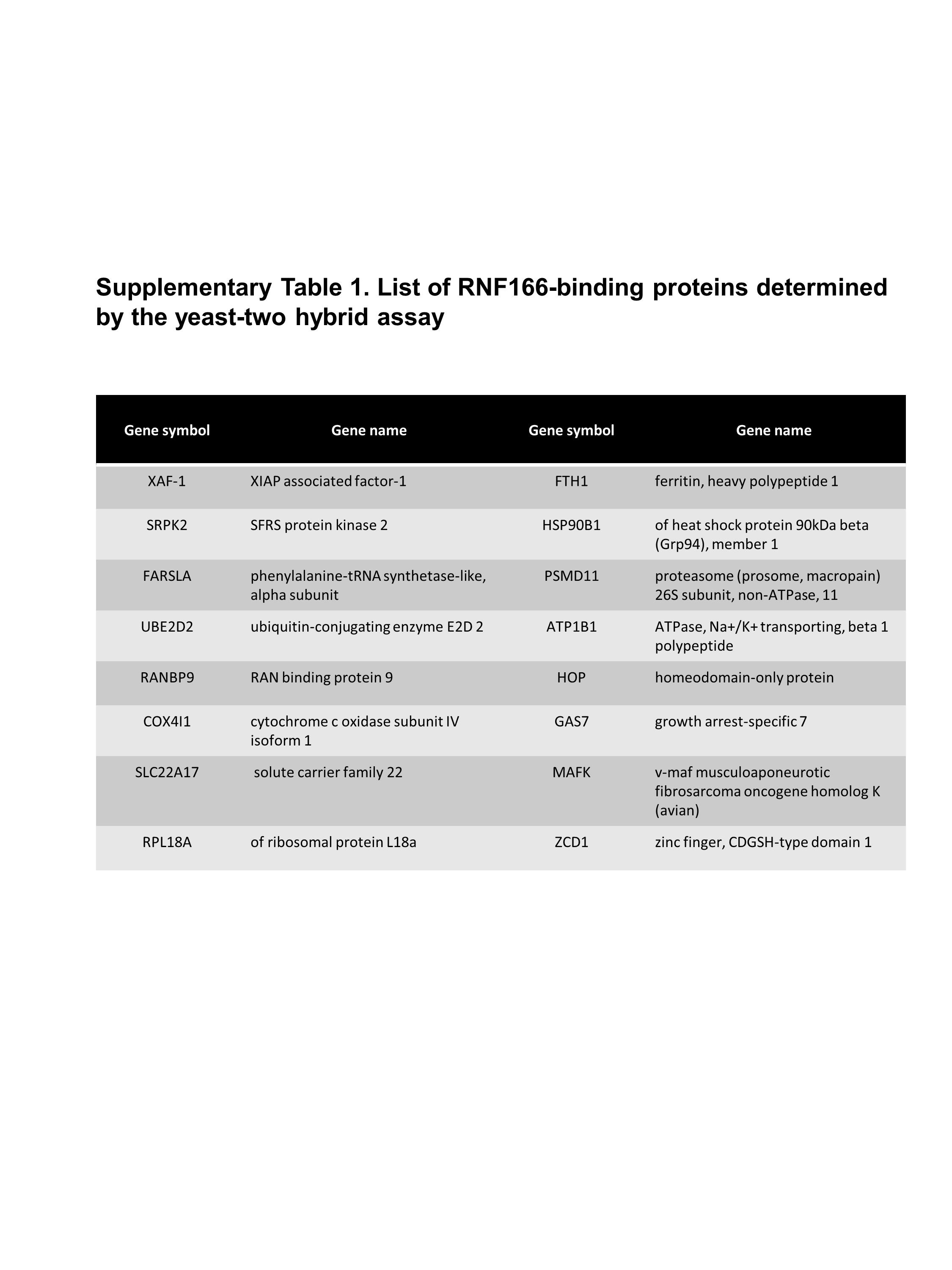

Supplement: Supplementary file 1 — Supplementary Table 1 [file 41419_2020_3145_MOESM1_ESM.tif]

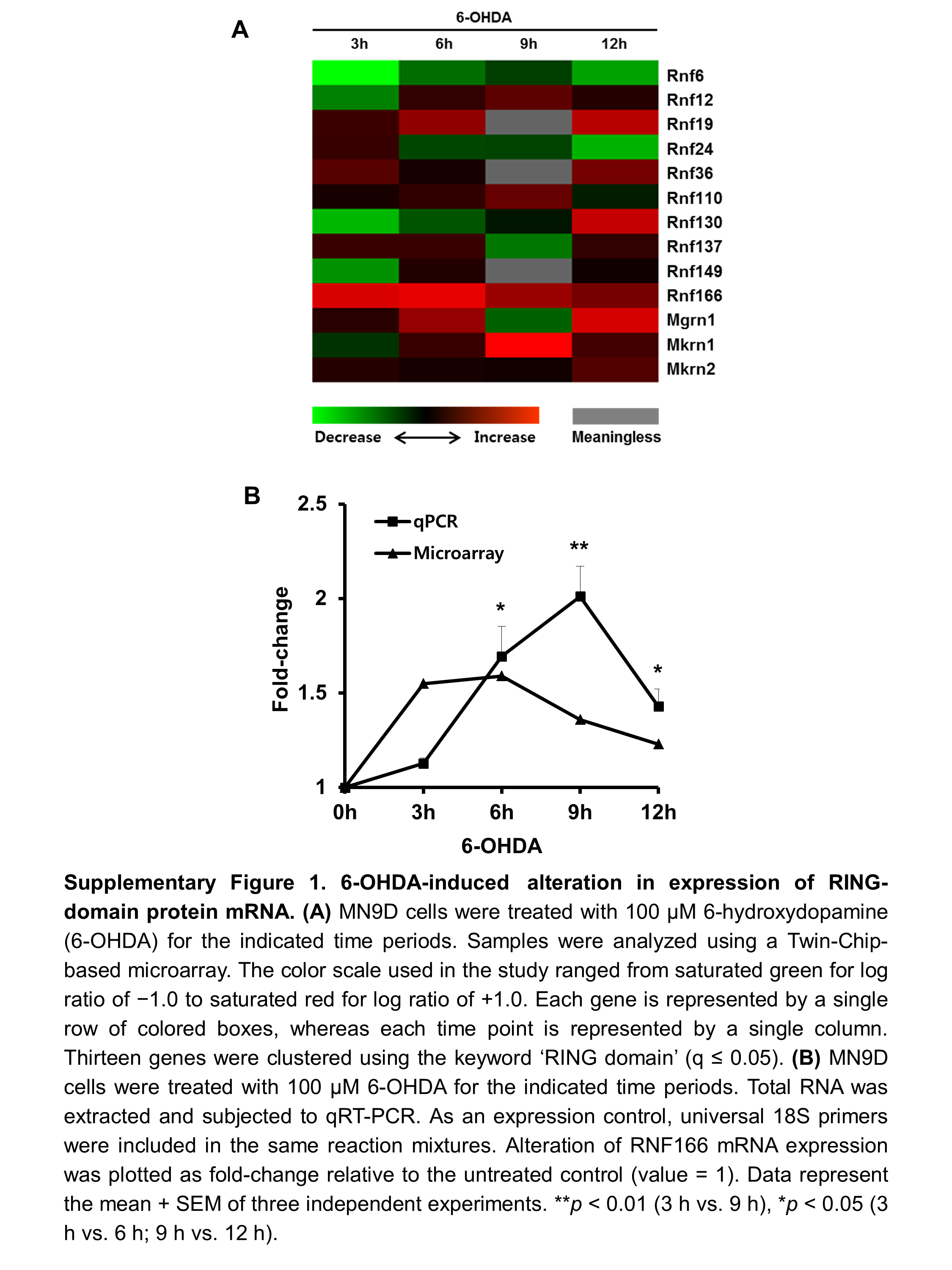

Supplement: Supplementary file 2 — Supplementary Figure 1 [file 41419_2020_3145_MOESM2_ESM.tif]

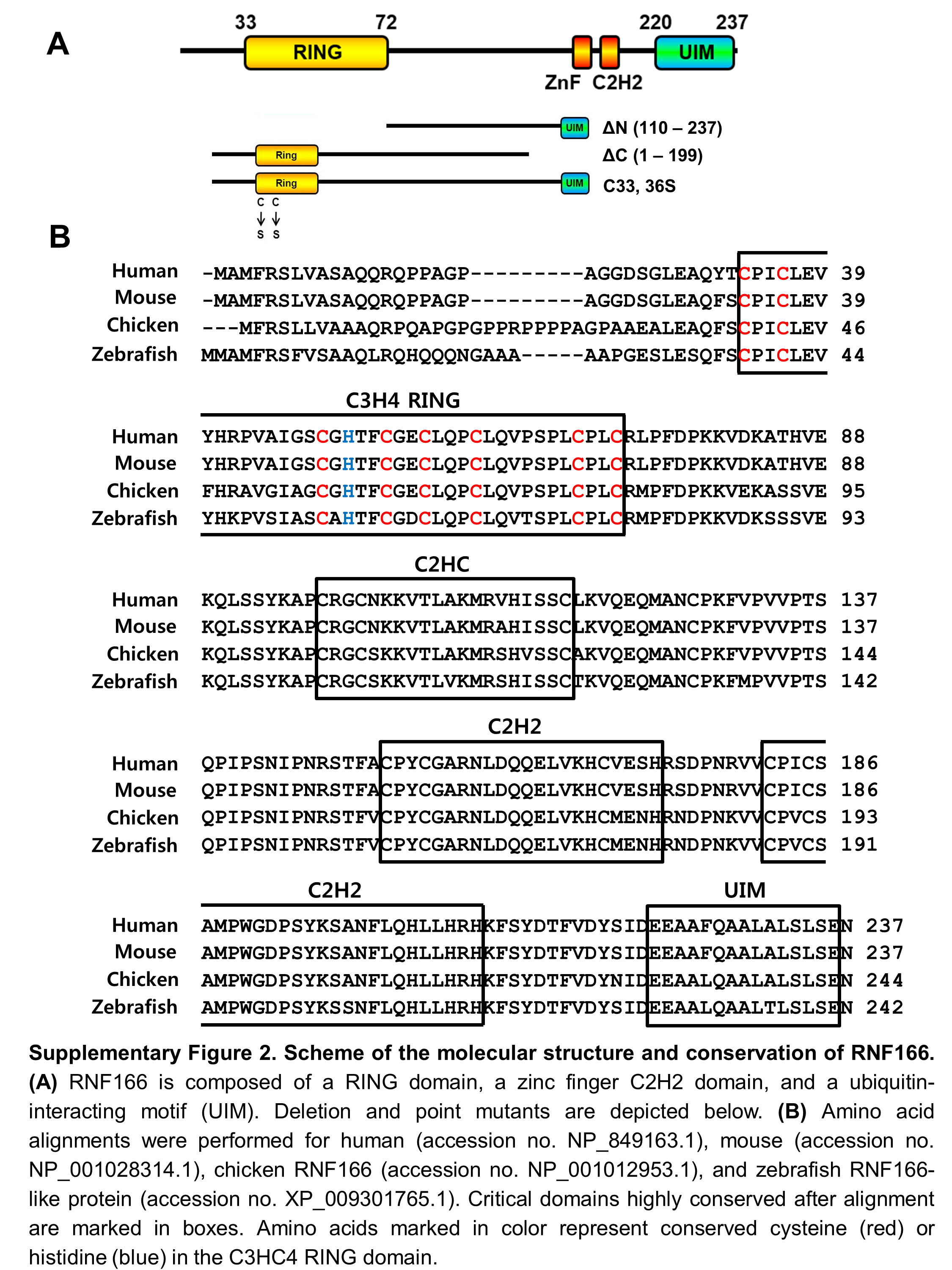

Supplement: Supplementary file 3 — Supplementary Figure 2 [file 41419_2020_3145_MOESM3_ESM.tif]

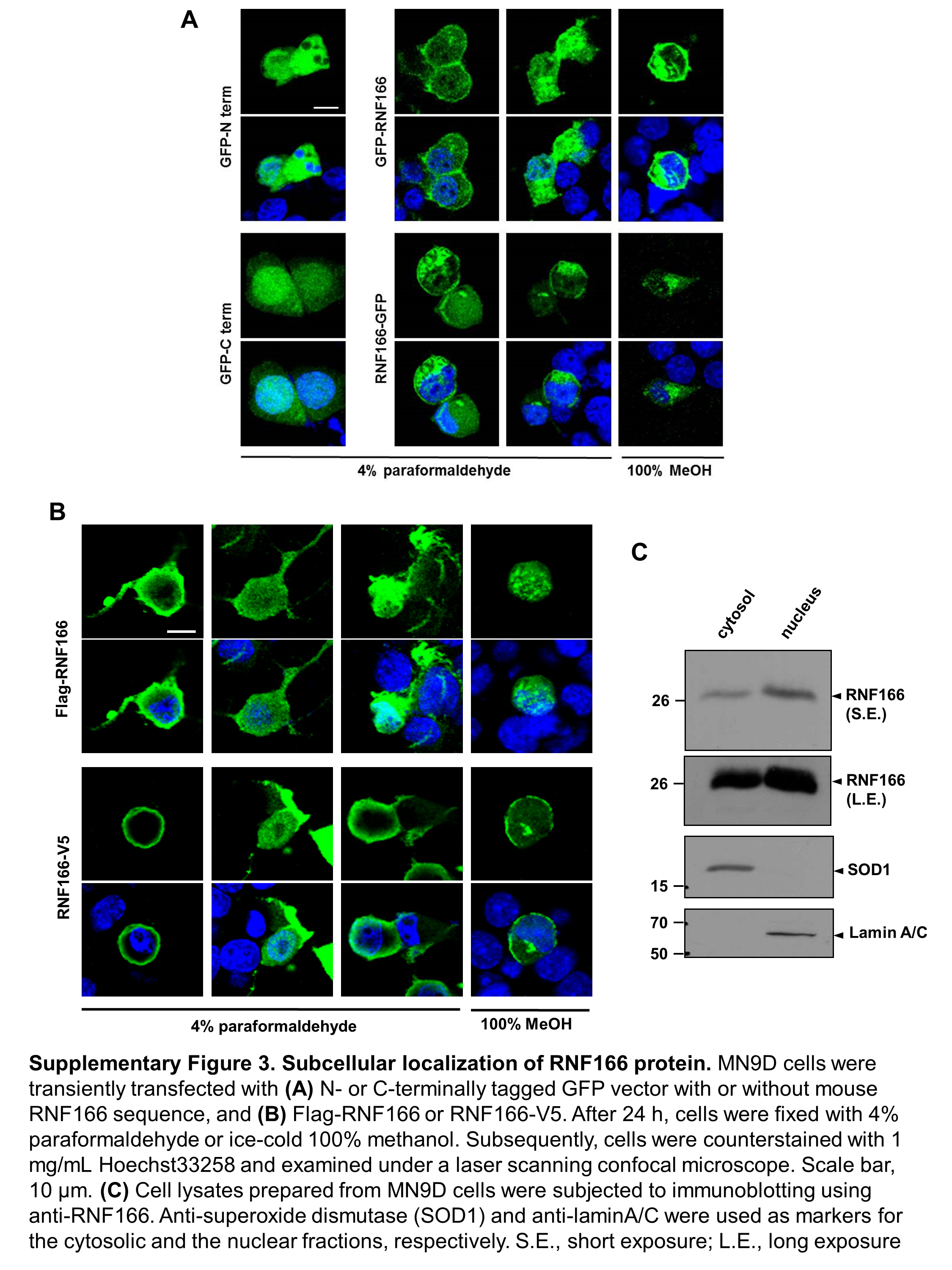

Supplement: Supplementary file 4 — Supplementary Figure 3 [file 41419_2020_3145_MOESM4_ESM.tif]

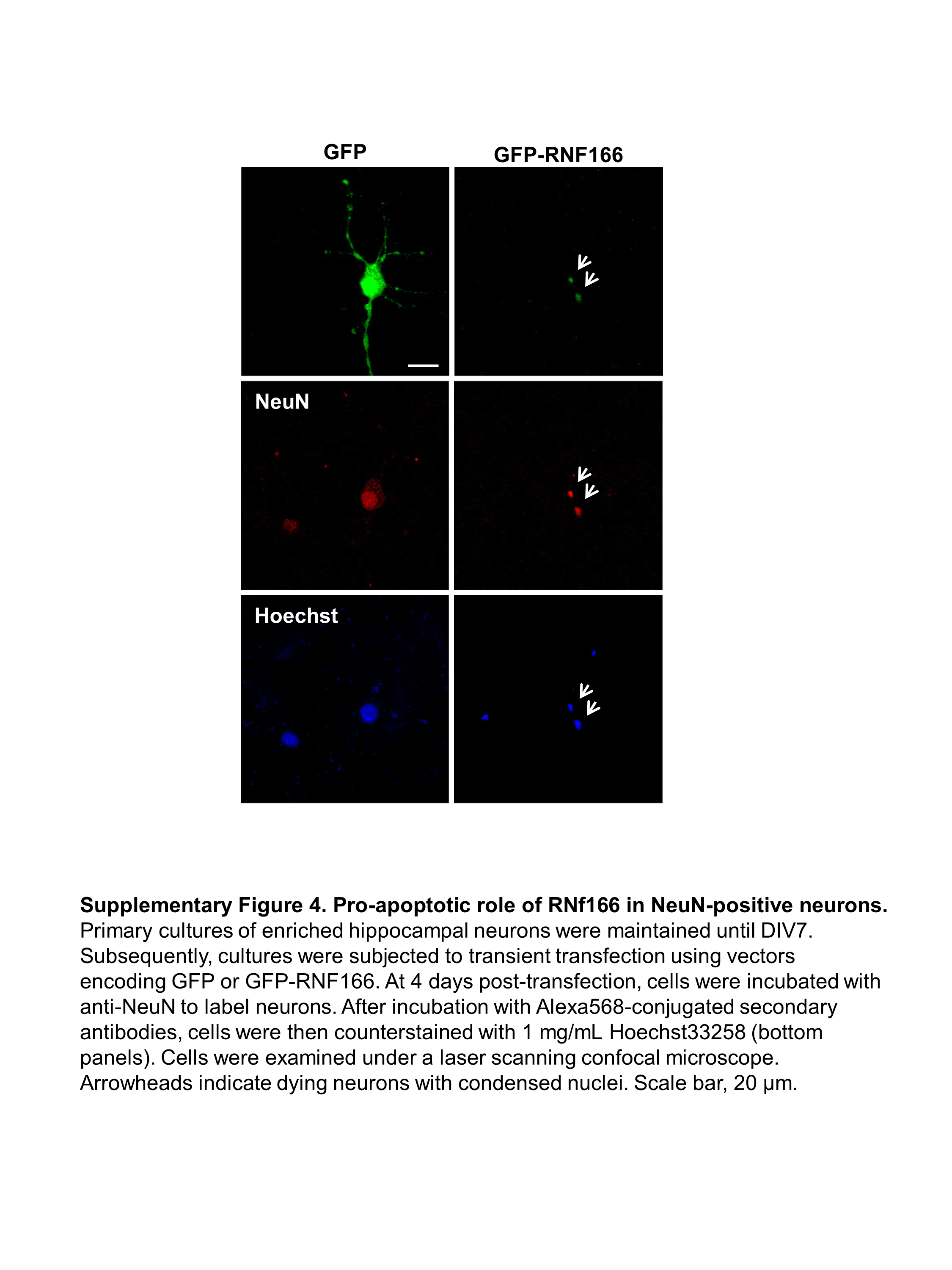

Supplement: Supplementary file 5 — Supplementary Figure 4 [file 41419_2020_3145_MOESM5_ESM.tif]

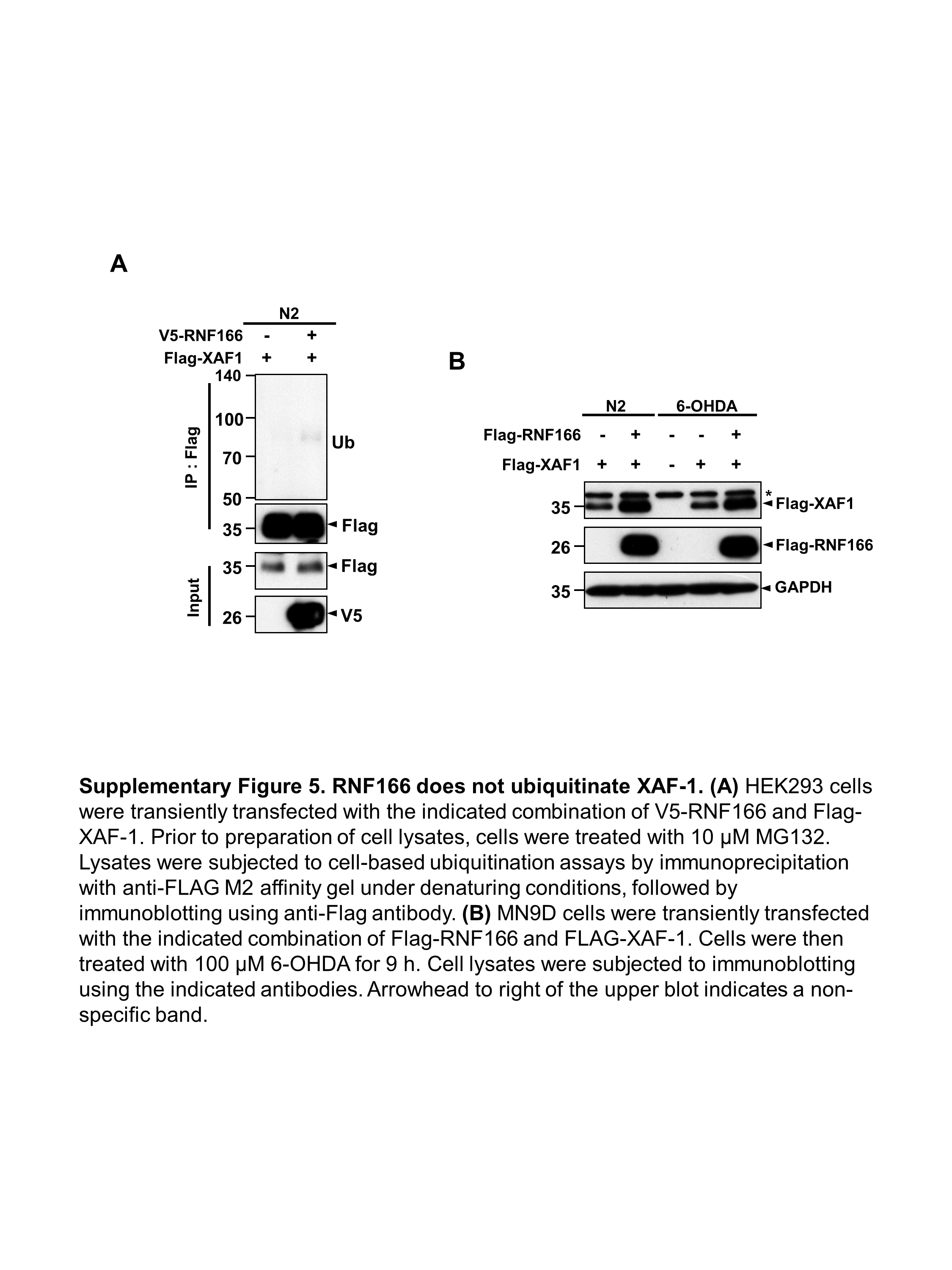

Supplement: Supplementary file 6 — Supplementary Figure 5 [file 41419_2020_3145_MOESM6_ESM.tif]

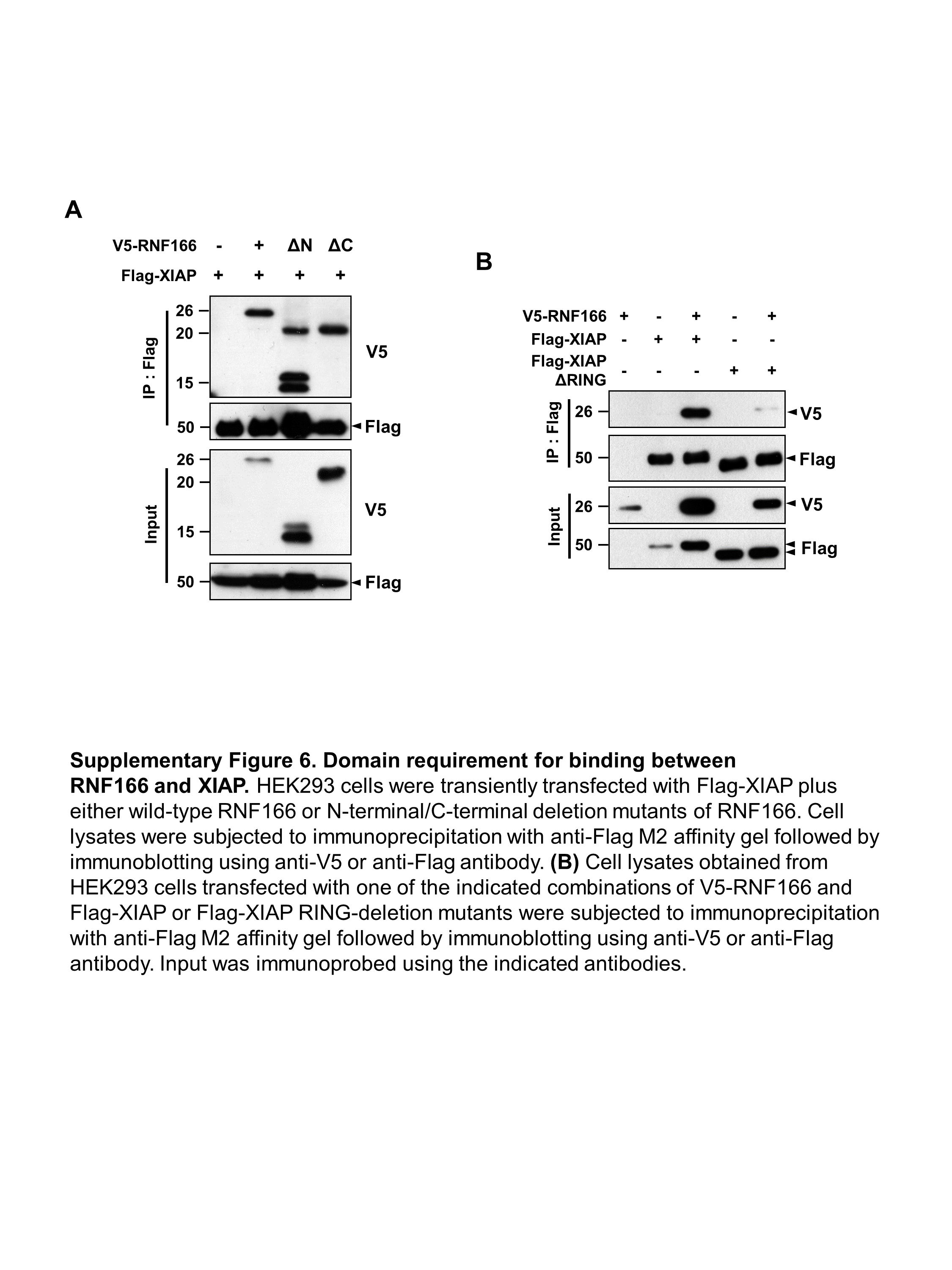

Supplement: Supplementary file 7 — Supplementary Figure 6 [file 41419_2020_3145_MOESM7_ESM.tif]

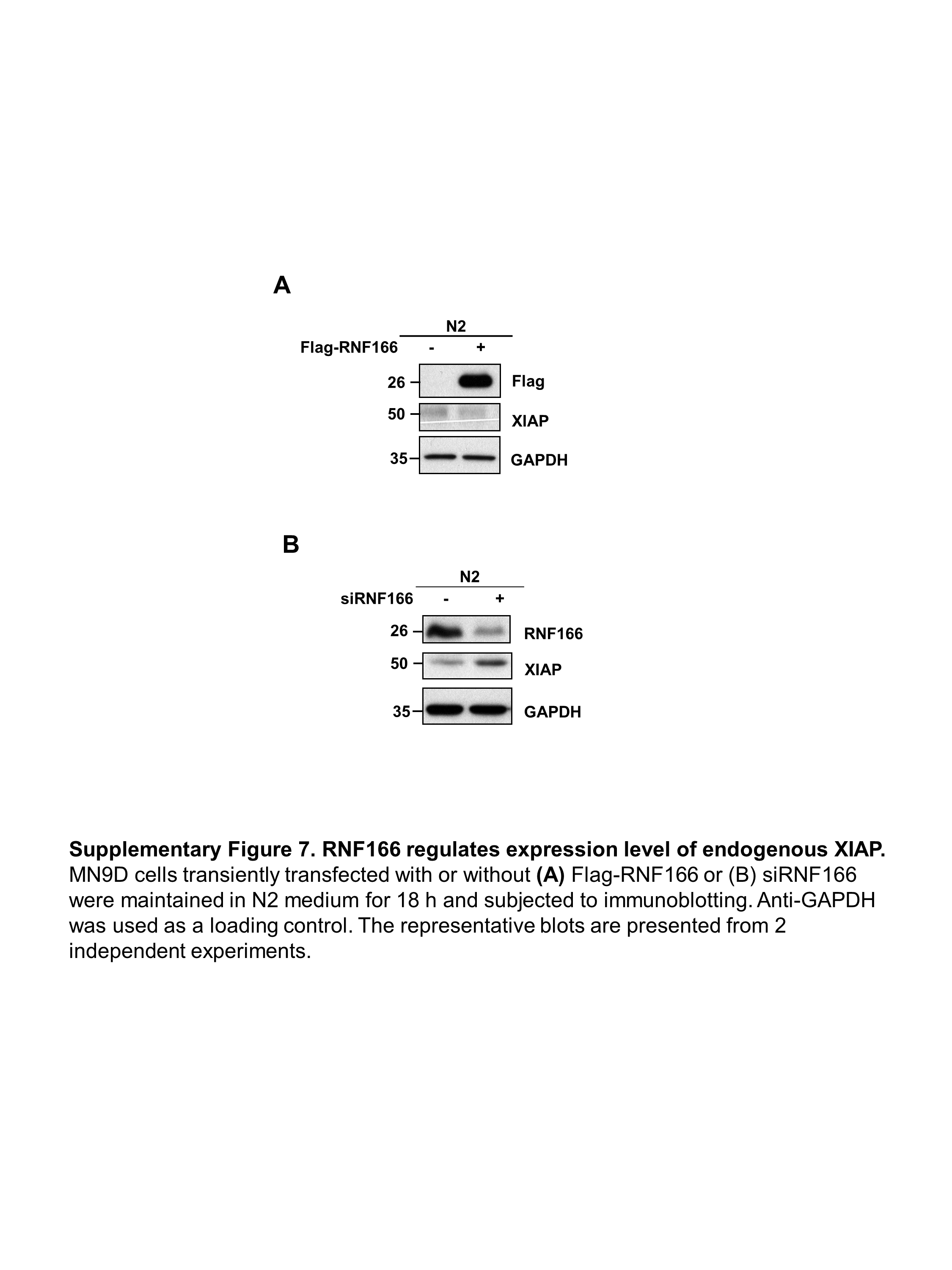

Supplement: Supplementary file 8 — Supplementary Figure 7 [file 41419_2020_3145_MOESM8_ESM.tif]

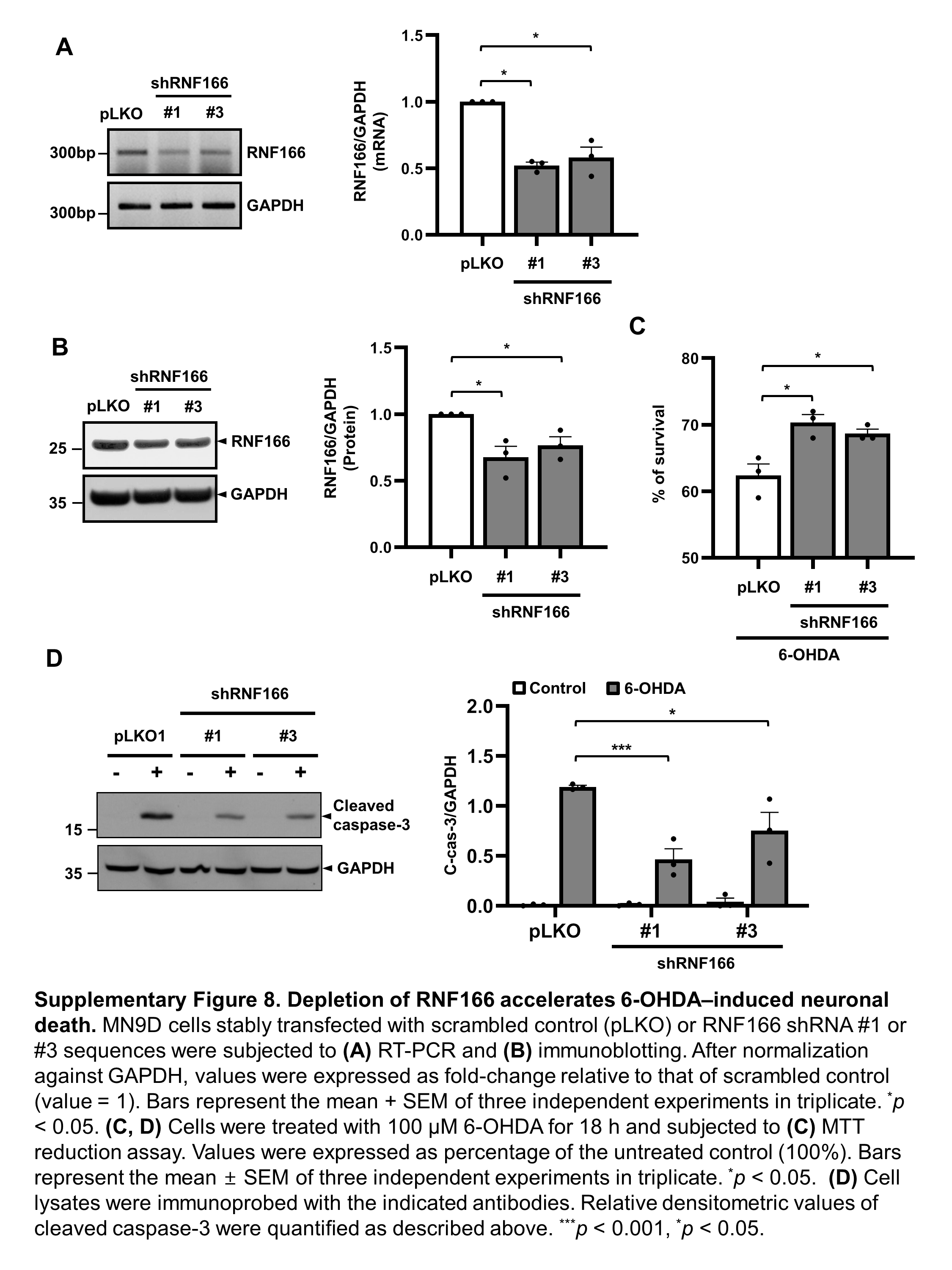

Supplement: Supplementary file 9 — Supplementary Figure 8 [file 41419_2020_3145_MOESM9_ESM.tif]

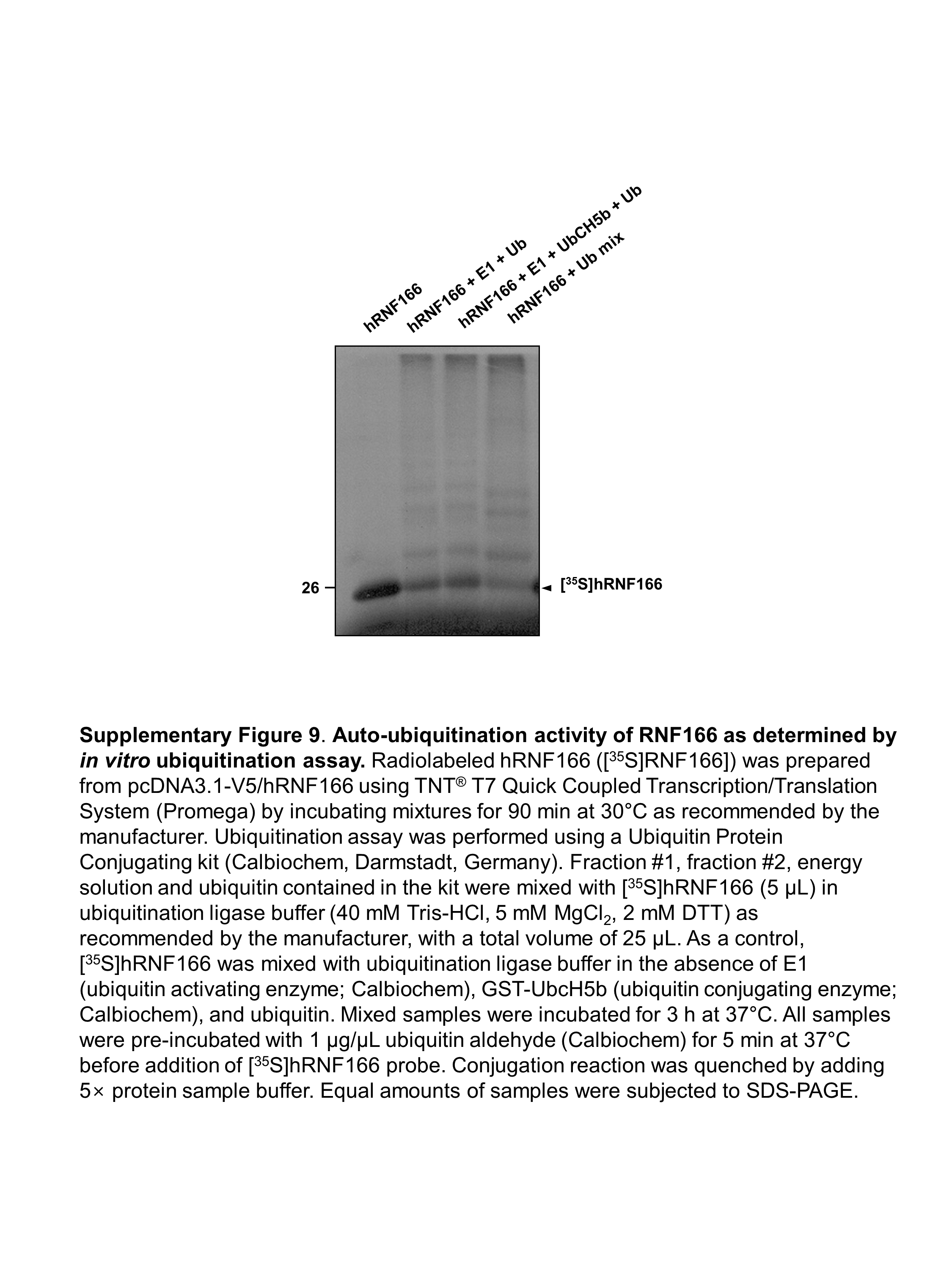

Supplement: Supplementary file 10 — Supplementary Figure 9 [file 41419_2020_3145_MOESM10_ESM.tif]

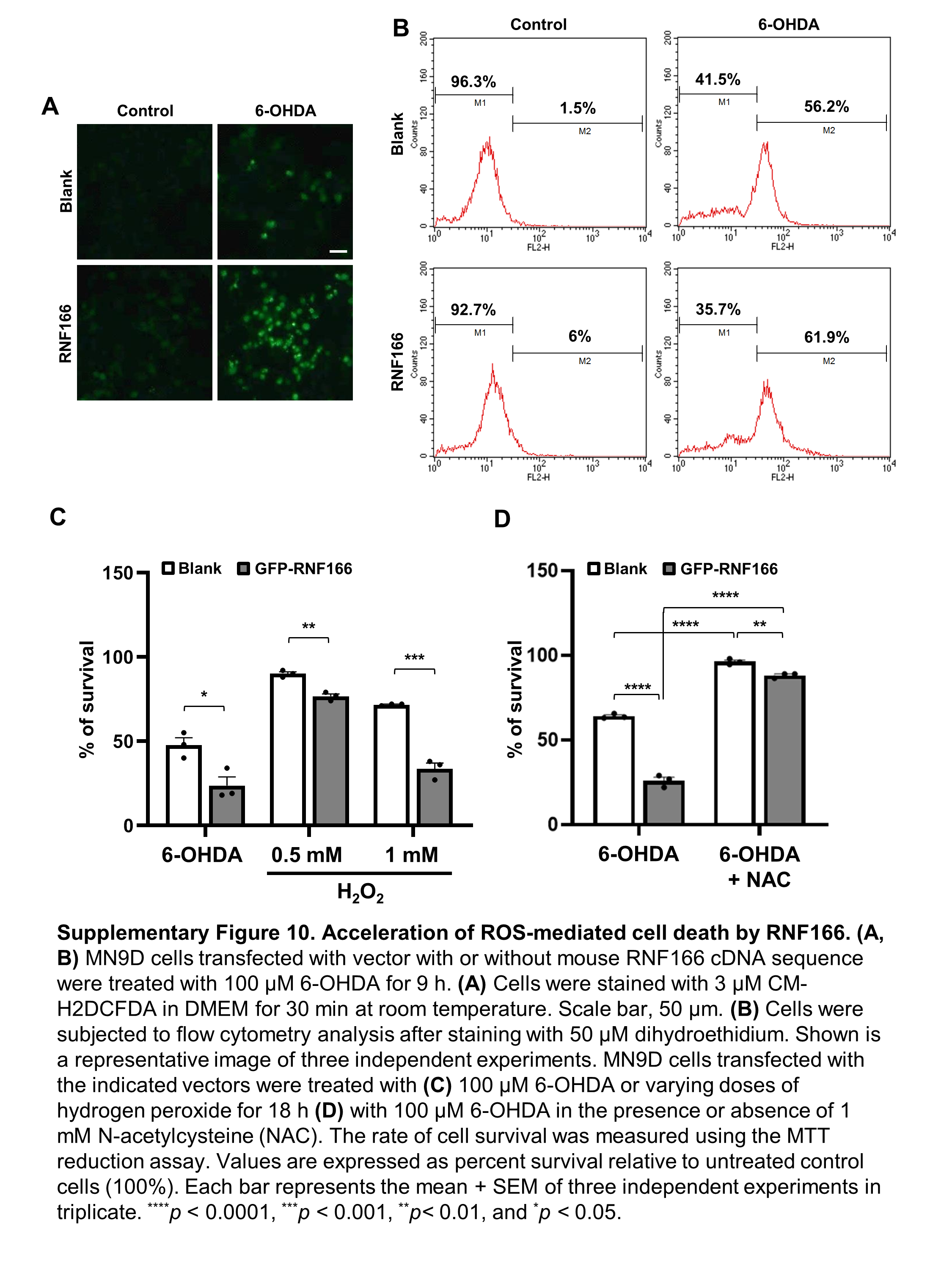

Supplement: Supplementary file 11 — Supplementary Figure 10 [file 41419_2020_3145_MOESM11_ESM.tif]
